# mTOR masters monocytic myeloid-derived suppressor cells in mice with allografts or tumors

**DOI:** 10.1038/srep20250

**Published:** 2016-02-01

**Authors:** Tingting Wu, Yang Zhao, Hao Wang, yang Li, Lijuan Shao, Ruoyu Wang, Jun Lu, Zhongzhou Yang, Junjie Wang, Yong Zhao

**Affiliations:** 1 State Key Laboratory of Membrane Biology, Institute of Zoology, Chinese Academy of Sciences, Beijing, China; 2 Department of Radiation Oncology, Peking University Third Hospital, Beijing, China; 3 Department of Oncology, the Affiliated Zhongshan Hospital of Dalian University, Dalian, China; 4 Hepatology and Cancer Biotherapy Ward, Beijing YouAn Hospital, Capital Medical University, Beijing, China; 5 MOE Key Laboratory of Model Animal for Disease Study, Model Animal Research Center, Nanjing University, Nanjing, China

## Abstract

CD11b^+^ Gr1^+^ myeloid-derived suppressor cells (MDSCs) play critical roles in controlling the processes of tumors, infections, autoimmunity and graft rejection. Immunosuppressive drug rapamycin (RPM), targeting on the key cellular metabolism molecule mTOR, is currently used in clinics to treat patients with allo-grafts, autoimmune diseases and tumors. However, the effect of RPM on MDSCs has not been studied. RPM significantly decreases the cell number and the immunosuppressive ability on T cells of CD11b^+^ Ly6C^high^ monocytic MDSCs (M-MDSCs) in both allo-grafts-transplanted and tumor-bearing mice respectively. Mice with a myeloid-specific deletion of mTOR have poor M-MDSCs after grafting with allo-skin tissue or a tumor. Grafting of allo-skin or tumors significantly activates glycolysis pathways in myeloid precursor cells in bone marrow, which is inhibited by RPM or mTOR deletion. 2-deoxyglucose (2-DG), an inhibitor of the glycolytic pathway, inhibits M-MDSC differentiation from precursors, while enhancing glycolysis by metformin significantly rescues the RPM-caused deficiency of M-MDSCs. Therefore, we offer evidence supporting that mTOR is an intrinsic factor essential for the differentiation and immunosuppressive function of M-MDSCs and that these metabolism-relevant medicines may impact MDSCs-mediated immunosuppression or immune tolerance induction, which is of considerable clinical importance in treating graft rejection, autoimmune diseases and cancers.

CD11b^+^ Gr1^+^ myeloid-derived suppressor cells (MDSCs) are now known to accumulate and play critical roles in various conditions like tumors, infections, autoimmune diseases and graft rejection^1^,^2^,^3^. These cells are a highly heterogeneous cell population with hematopoietic cell precursors at various differentiation stages to mature macrophages, dendritic cells (DCs), and granulocytes^4^. In general, MDSCs are divided into monocytic (M-MDSCs, CD11b^+^ Ly6C^high^) and granulocytic (G-MDSCs, CD11b^+^ Ly6C^medium^) subpopulations, which are distinguished phenotypically^1^,^5^,^6^. MDSCs-mediated suppression on T cells through multiple molecular mechanisms. High levels of both arginase 1 (Arg1) and inducible nitric oxide synthase (iNOS) expressed by MDSCs resulted in depletion of L-Arginine in the microenvironment which is essential for T cell proliferation^7^. Reactive oxygen species (ROS) of MDSCs via catalyze the nitration of TCR, which consequently decreases the T cell-peptide/MHC interaction^8^. In addition, other mechanisms mediated by heme oxygenase-1 (HO-1), indoleamine 2,3 dioxygenase (IDO) and membrane-bound TGF-β1 *et al.* are also involved in MDSCs-mediated immunosuppression in certain cases have been reported^9^,^10^,^11^,^12^. It is demonstrated that signal transducer and activator of transcription (STAT3, STAT1, STAT5 and STAT6) and NF-κB may promote the differentiation of MDSCs^13^, whereas Smad3 negatively regulates CD11b^+^ Gr1^+^ MDSC maturation and function^14^. However, the intrinsic molecular mechanisms for controlling CD11b^+^ Gr1^+^ MDSC differentiation and function are still poorly understood.

The mammalian target of rapamycin (RPM) (mTOR) pathway is well recognized to master cell metabolism, proliferation and survival. The specific inhibitor of mTOR, RPM, is widely used in clinics to treat allograft rejection, autoimmune diseases and some cancers today^15^,^16^,^17^,^18^. In addition to its efficient effects on T cell subsets^19^,^20^,^21^, RPM has recently emerged as an important regulator of innate immune cell homeostasis and inflammatory response^22^,^23^,^24^,^25^. However, whether mTOR pathway is involved in MDSC induction and function in settings of transplantation and tumors needs to be addressed. In the present study, we investigated the effects of RPM on MDSCs in allogeneic skin (alloskin)-grafted mice and tumor-bearing mice respectively. Our results show that RPM treatment significantly decreases CD11b^+^ Ly6C^high^ M-MDSCs but not G-MDSCs in these two experimental models. Furthermore, studies using mice with a myeloid-specific deletion of mTOR or mTORC2 essential component rictor demonstrate that mTORC1 but not mTORC2 intrinsically controls CD11b^+^ Ly6C^high^ M-MDSC differentiation and immunosuppressive function through controlling cellular metabolism pathway. Moreover, blocking glycolysis by 2-deoxyglucose (2-DG) decreased M-MDSC differentiation and enhancing glycolysis by metformin promotes M-MDSC differentiation. Therefore, our study suggests that RPM and 2-DG treatment may potentially block MDSCs-mediated immune tolerance establishment in transplant settings and likely promote anti-tumor immune response in terms of regulation on MDSCs. On the other hand, metformin potentially promotes M-MDSCs-mediated immune down-regulation or tolerance. We believe the present study may have great potential impacts on the clinical usage of RPM, 2-DG and metformin to treat patients with allograft transplantation, autoimmune diseases and cancers.

## Results

### RPM significantly decreases M-MDSCs in alloskin-grafted mice

**Figure 1:**
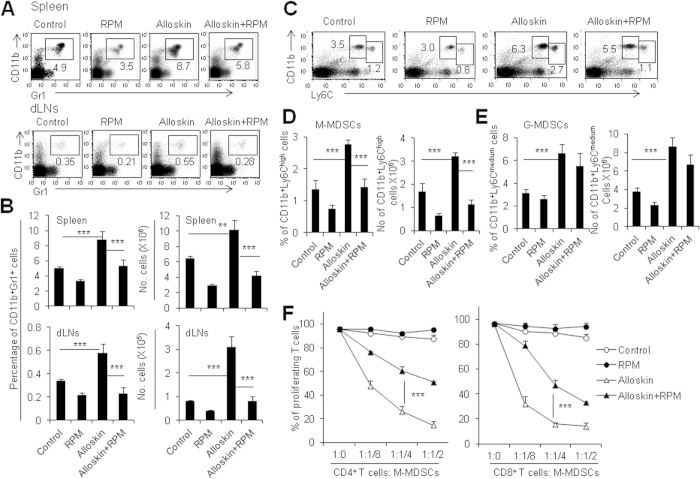
RPM significantly decreases M-MDSCs in alloskin-grafted mice. Cells of spleens and draining lymph nodes (dLNs) from control and alloskin-grafted mice treated with or without RPM at day 7 after skin grafting were stained with anti-CD11b and anti-Gr-1 mAbs. (**A**) Typical example of flow cytometry analysis. The numbers in FACS plots were the percentages of CD11b^+^ Gr1^+^ cells in spleens and dLNs. (**B**) The percentages and total cell numbers of CD11b^+^ Gr-1^+^ cells in spleens and dLNs from the four groups of mice are summarized. Splenocytes were stained with anti-CD11b and anti-Ly6C antibodies. (**C**) Typical example of flow cytometry analysis. M-MDSCs were gated as CD11b^+^ Ly6C^high^ cell subset and G-MDSCs were gated as CD11b^+^ Ly6C^medium^ cell subset. The numbers in FACS plots were the percentages of cells in the indicated gate. The percentages (**D**) and cell numbers (**E**) of M-MDSCs and G-MDSCs in spleens from the four groups of mice as indicated. (**F**) Naive splenocytes (2 × 10^5^/well) were pre-labeled with CFSE and activated with anti-CD3 (1μg/ml) and anti-CD28 (5μg/ml) mAbs in the presence of the freshly isolated CD11b^+^ Ly6C^hi^ cells from spleens of recipients at the indicated ratios. Cells were co-cultured for 72 h, and CD4^+^ and CD8^+^ T cell proliferation was measured by CFSE dye dilution. Results are expressed as mean ± SD. Each group included from 5 to 8 mice and minimum three independent experiments showed similar results. **p < 0.01, and ***p < 0.001 compared between the indicated groups.

In order to understand the effects of RPM on allograft-induced MDSCs, we first employed the alloskin-grafted mouse model. RPM-treated B6 mice (H-2^b^) rejected BALB/c (H-2^d^) alloskin grafts at a significantly increased median survival time (MST) as long as 13 days, whereas the control B6 mice rejected alloskin grafts with a MST of about 10 days as expected (p < 0.05, Suppl. Fig. 1A). Alloskin grafts promoted the accumulation of CD11b^+^ Gr1^+^ innate cells in the spleens and dLNs of recipient mice compared with the un-grafted mice (P < 0.001, Fig. 1A,B), as reported previously^3^,^14^. However, RPM treatment significantly blocked the induction of CD11b^+^ Gr1^+^ innate cells by alloskin grafts in the spleens and dLNs of recipient mice in respect of percentages and total cell numbers (P < 0.001, Fig. 1A,B). It is known that CD11b^+^ Gr1^+^ MDSCs consists of two major subsets of granulocytic CD11b^+^ Ly6C^med^ and monocytic CD11b^+^ Ly6C^high^ cells^26^. We therefore assayed these two subsets in the alloskin-grafted mice using anti-Ly6C and anti-CD11b mAbs. RPM selectively decreased the percentages and cell numbers of CD11b^+^ Ly6C^high^ innate cells in the spleens and dLNs of alloskin-grafted mice (P < 0.001, Fig. 1C,D), while the CD11b^+^ Ly6C^med^ granulocytic cells were not significantly impacted by RPM in these mice (Fig. 1C,E). The percentage of CD11b^+^ Ly6C^high^ innate cells in the alloskin grafts of RPM-treated mice was also remarkably decreased compared to alloskin-grafted mice (Suppl. Fig. 1B,C), while the CD11b^+^ Ly6C^med^ granulocytic cells in alloskin grafts were weakly impacted by RPM in these mice (Suppl. Fig. 1D). Additionally, we also found that CD4 and CD8 T cells were decreased in alloskin grafts of RPM-treated mice (Suppl. Fig. 2). The decreased T cells in alloskin grafts are likely due to the direct inhibitory effects of RPM on T cells in recipient mice. To further define whether Ly6C^high^ population belongs to monocytic cells, we observed the Ly6G expression on the gated Ly6C^high^ and Ly6C^medium^ cells. As shown in Suppl. Fig. 3, almost no cells in the Ly6C^high^ cell population express Ly6G, whereas a high percentage of Ly6C^medium^ cells express Ly6G (Suppl. Fig. 3A). These data indicate that Ly6C^high^ cells are mainly monocytic, while Ly6C^medium^ cells mainly belong to granulocyte population. Furthermore, the percentages of CD11b^+^ Ly6C^high^Ly6G^−^ and CD11b^+^ Ly6C^medium^Ly6G^+^ subpopulations were observed using multiple-color staining flow cytometry. RPM treatment significantly decreased CD11b^+^ Ly6C^high^Ly6G^−^ monocytic cell population but no detectable effects on CD11b^+^ Ly6C^medium^Ly6G^+^ subpopulation (Suppl. Fig. 3B). Thus, RPM selectively impacts on CD11b^+^ Ly6C^high^Ly6G^−^ monocytic cell population in alloskin-grafted mice. To determine whether the accumulated CD11b^+^ Ly6C^high^ cells in alloskin-grafted mice are so-called immunosuppressive MDSCs, we performed immunosuppression assays using T cells as effectors as reported previously^14^. It is true that CD11b^+^ Ly6C^high^ cells isolated from skin-grafted mice displayed dose-dependent inhibitory effects on both CD4^+^ and CD8^+^ T cell proliferation (Fig. 1F, Suppl. Fig. 4). However, CD11b^+^ Ly6C^medium^ cells isolated from RPM-treated skin-grafted mice failed to efficiently inhibit T cell proliferation (Suppl. Fig. 5). Thus, RPM treatment selectively inhibits the immunosuppressive CD11b^+^ Ly6C^high^Ly6G^−^ M-MDSC induction and immunosuppressive function in alloskin-grafted recipients.

### Deletion of mTOR in myeloid cells decreases M-MDSCs in mice after transplantation

**Figure 2:**
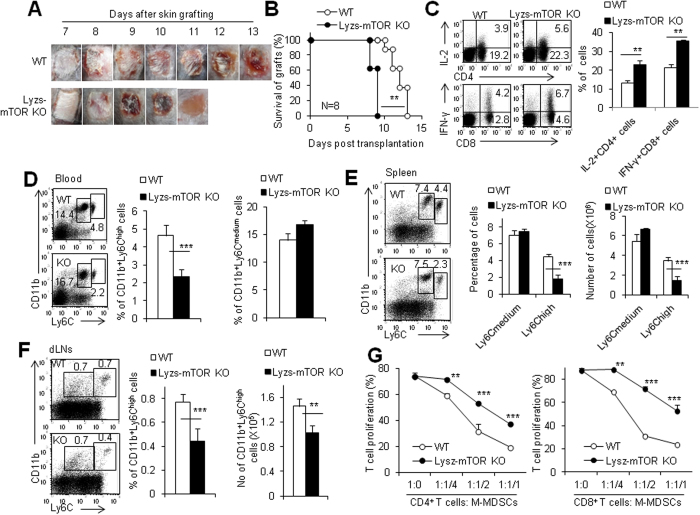
Deletion of mTOR specifically in myeloid cells decreases M-MDSCs in mice after transplantation. (**A**) Age and gender matched WT and lyzs-mTOR KO mice were transplanted with allogeneic BALB/c tail skin and macroscopic pictures of alloskin grafts were collected at different time points. (**B**) Alloskin graft rejection in lyzs-mTOR KO recipients was significantly accelerated compared with WT recipients. The splenocytes, peripheral blood cells and draining lymph node cells of alloskin-grafted WT and Lyzs-MTOR KO recipients were isolated and analyzed by flow cytometry at day 7 after skin grafting. (**C**) The expression of IFN-γ and IL-2 proteins in gated CD4^+^ T cells and CD8^+^ T cells in draining lymph node of WT and lyzs-mTOR KO recipients as determined by intracellular staining. (**D**) The percentages of CD11b^+^ Ly6C^hi^ M-MDSCs and CD11b^+^ Ly6C^med^ G-MDSCs in peripheral blood of WT and lyzs-mTOR KO recipients. The percentages and cell numbers of CD11b^+^ Ly6C^hi^ M-MDSCs and CD11b^+^ Ly6C^med^ G-MDSCs in spleens (**E**) and dLNs (**F**) of WT and lyzs-mTOR KO recipients. (**G**) Isolated CD11b^+^ Ly6C^hi^ cells from spleens of WT and lyzs-mTOR KO recipients at day 7 after skin grafting were added at different ratios in T cell proliferation system for 72 h. Proliferation was measured by CFSE dye dilution. The numbers in FACS plots represent the percentages of cells in the gates. Results are expressed as mean ± SD. Each group included from 4 to 6 mice and at least three independent experiments showed similar results. **p < 0.01, and ***p < 0.001 compared between the indicated groups.

It is well known that RPM acts on mTOR and modulates many immune cell subpopulations such as T cells and macrophages in a positive or negative way^24^,^25^,^27^,^28^. To address whether RPM acts on CD11b^+^ Ly6C^high^ M-MDSCs directly or indirectly, we employed mice with a myeloid-specific deletion of mTOR (Suppl. Fig. 6) as recipients to exclude the indirect effects on M-MDSCs of RPM due to the direct actions on T cells of RPM which certainly occur in RPM-treated mice. The deletion efficiency of mTOR in the sorted monocytes/macrophages and granulocytes was determined by real-time PCR, western blots and mTOR activity indicated by p-S6 (Suppl. Fig. 6). On the other hand, Lyzs was mainly expressed in CD11b^+^ Ly6C^high/medium^ cells, whereas almost no CD11b^+^ CD11c^+^ dendritic cells express Lyzs, as determined by Lyzs-GFP reporter mice (Suppl. Fig. 7). Thus, Lyzs-cre/mTOR loxp mice selectively delete mTOR gene in CD11b^+^ Ly6C^+^ cells but not CD11c^+^ cells. With Lyzs-mTOR KO mice as recipients of alloskin grafts, we observed the skin graft survival and detected the levels of immune cell subpopulations. As shown in Fig. 2, from day 7 to 11, alloskin grafts on WT recipients showed progressing loss of hair, dermal necrosis and scab formation. However, the alloskin grafts on Lyzs-mTOR KO recipients showed more aggressive necrosis and scab formation as early as day 8 after grafting (Fig. 2A). In line with these observations, the skin grafts were fully rejected by Lyzs-mTOR KO mice more rapidly than by WT mice (P < 0.01, Fig. 2B), which is in contrast to the observation showing that RPM treatment significantly prolonged alloskin graft survival. To determine the intensity of adaptive immunity to allo-grafts in Lyzs-mTOR KO recipients, we detected the levels of anti-donor IgG and IgG2b subclasses in the serum and the expressions of inflammatory cytokines like IL-6 and IFN-γ in CD4^+^ and CD8^+^ T cells by flow cytometry assays as described previously^29^. Significantly higher percentages of CD4^+^ and CD8^+^ T cells of Lyzs-mTOR KO recipients expressed IL-2 and IFN-γ than those in WT recipients respectively (P < 0.01, N = 5, Fig. 2C). In parallel, the levels of total anti-donor IgG and IgG2b in Lyzs-mTOR KO recipients were significantly higher than WT recipients after 2 weeks post transplantation (Suppl. Fig. 8). Therefore, these data collectively indicate that recipients with an mTOR deficiency specifically in myeloid cells promoted an enhanced adaptive immunity against alloskin grafts.

Meanwhile, significantly less percentages of CD11b^+^ Ly6C^high^ monocytic cells but not CD11b^+^ Ly6C^medium^ granulocytic cells in the peripheral blood of skin-grafted Lyzs-mTOR KO recipients were observed compared to WT recipients (P < 0.001, Fig. 2D). CD11b^+^ Ly6C^high^ monocytic cells in the spleens of Lyzs-mTOR KO recipients were significantly less than in WT recipients in terms of the percentages and total cell numbers (P < 0.001, Fig. 2E). The levels of CD11b^+^ Ly6C^medium^ granulocytic cells were identical in spleens of WT and Lyzs-mTOR KO recipients (P > 0.05, Fig. 2E). In addition, there were less CD11b^+^ Ly6C^high^ monocytic cells in the dLNs and alloskin grafts of skin-grafted Lyzs-mTOR KO recipients (P < 0.01, Fig. 2F, Suppl. Fig. 9A). Furthermore, when the immunosuppressive function was evaluated using the standard immunosuppression assays *in vitro*, CD11b^+^ Ly6C^high^ monocytic cells isolated from alloskin-grafted Lyzs-mTOR KO mice showed poor immunosuppressive ability on T cell proliferation compared to those from WT recipients (P < 0.001, Fig. 2G). In consistent with the *in vitro* data, more CD4^+^ and CD8^+^ T cells in alloskin grafts in Lyzs-mTOR KO mice than those in WT recipients was observed (P < 0.05, Suppl. Fig. 9B). Thus, deletion of mTOR in myeloid cells selectively blocked the differentiation of CD11b^+^ Ly6C^high^ M-MDSC subpopulations in alloskin-transplanted mice. The poor CD11b^+^ Ly6C^high^ M-MDSCs may be one of the reasons for the accelerated alloskin graft rejection in Lyzs-mTOR KO mice.

### Decreased tumor-induced MDSCs in mice with a myeloid-specific deletion of mTOR

**Figure 3:**
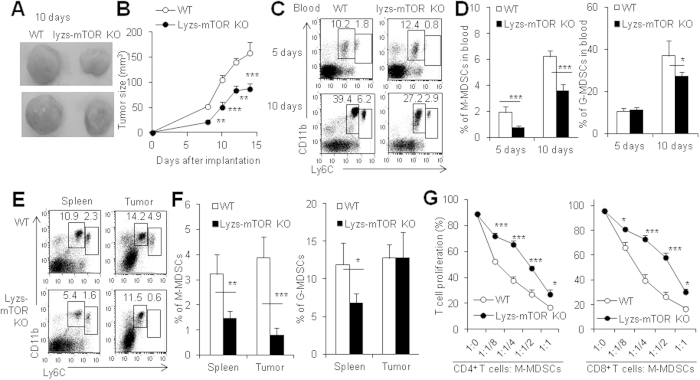
Decreased tumor-induced MDSCs in mice with a myeloid-specific deletion of mTOR. WT and lyzs-mTOR KO mice with the same age and gender were injected s.c. with 1.5 × 10^6^ EL4 lymphoma. (**A**) Macroscopic pictures of implanted tumor in WT and lyzs-mTOR KO mice at day 10 after implantation. (**B**) The tumor size was measured from day 5 to day 15, when tumors reached a maximum. (**C**) The representative of CD11b and Ly6G staining of white blood cells. (**D**) The percentages of CD11b^+^ Ly6C^hi^ M-MDSCs and CD11b^+^ Ly6C^med^ G-MDSCs in peripheral blood of WT and lyzs-mTOR KO mice at day 5 and day 10 after tumor grafting. (**E**) The representative of CD11b and Ly6G staining of cells isolated from spleens and tumors. (**F**) The percentages of CD11b^+^ Ly6C^hi^ M-MDSCs and CD11b^+^ Ly6C^med^ G-MDSCs in spleens and tumor mass of WT and lyzs-mTOR KO mice at day 10 after tumor implantation. (**G**) Isolated CD11b^+^ Ly6C^hi^ cells from spleens of WT and lyzs-mTOR KO mice at day 10 after tumor grafting were added at different ratios in T cell proliferation system for 72h. Cell proliferation was measured by CFSE dye dilution. The numbers in FACS plots represent the percentages of cells in the gates. Results are expressed as mean ± SD. Each group included from 6 to 8 mice and minimum three independent experiments showed similar results. **p < 0.01, and ***p < 0.001 compared between the indicated groups.

To investigate whether the essential role of mTOR in M-MDSC differentiation is limited in allo-graft transplantation settings alone or is a general phenomenon which occurs in other MDSC inducing situations such as tumors, we used tumor-bearing mouse models. RPM treatment obviously decreased CD11b^+^ Ly6C^high^ monocytic cells in the peripheral blood and spleens of B16- or EL4-bearing B6 mice (Suppl. Fig. 10). Importantly, EL4 tumor growth is significantly slower in Lyzs-mTOR KO mice than in WT recipients (P < 0.01, Fig. 3A,B). The percentages of CD11b^+^ Ly6C^high^ monocytic cells in the peripheral blood, spleens and tumor mass of Lyzs-mTOR KO mice were significantly lower than those in WT recipients (P < 0.001, Fig. 3C–F). Consistently, CD11b^+^ Ly6C^high^Ly6G^−^ monocytic cells but not CD11b^+^ Ly6C^medium^Ly6G^+^ granulocytic cells in tumor-bearing Lyzs-mTOR KO mice were significantly less than in tumor-bearing WT mice (Suppl. Fig. 11). Functional assays showed that CD11b^+^ Ly6C^high^ monocytic cells of tumor-bearing Lyzs-mTOR KO mice had significantly less immunosuppressive than those cells of WT recipients (P < 0.01, Fig. 3G, Suppl. Fig. 12). The poor immunosuppressive function of CD11b^+^ Ly6C^high^Ly6G^−^ monocytic cells in tumor-bearing Lyzs-mTOR KO mice was indirectly supported by the increased infiltration of CD4^+^ and CD8^+^ T cells in the tumor tissue of Lyzs-mTOR KO mice compared with those in WT mice (Suppl. Fig. 13). Therefore, inhibition or deletion of mTOR in myeloid cells caused a defect CD11b^+^ Ly6C^high^Ly6G^−^ M-MDSC differentiation in tumor-bearing recipients.

### RPM directly inhibits M-MDSC differentiation *in vitro*

**Figure 4:**
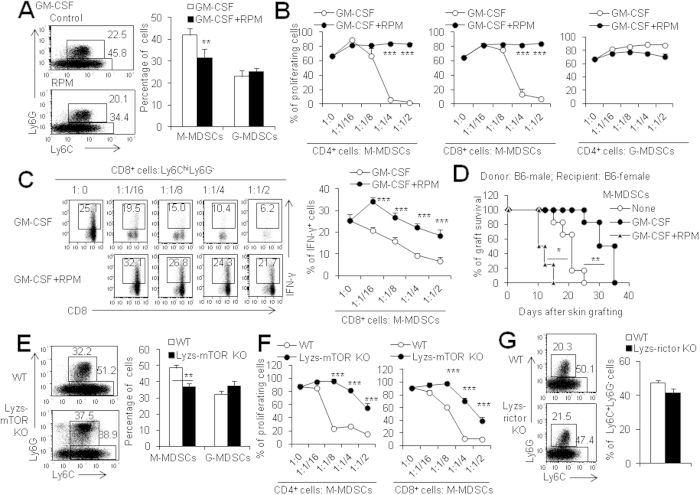
RPM directly inhibits M-MDSC differentiation *in vitro.* MDSCs induced from BM cells by GM-CSF with or without 1 μM RPM for 4 days *in vitro*. (**A**) Left panel: typical example of flow cytometry analysis. Ly6C^hi^Ly6G^−^ cells were defined as M-MDSCs and Ly6C^med^Ly6G^+^ cells as G-MDSCs. Right panel: the percentage of M-MDSCs and G-MDSCs. (**B**) T cell proliferation in the presence of the sorted CD11b^+^ Ly6C^hi^Ly6G^−^ M-MDSCs and CD11b^+^ Ly6C^med^Ly6G^+^ G-MDSCs was measured by CFSE dye dilution. (**C**) Sorted CD11b^+^ Ly6C^hi^Ly6G^−^ M-MDSCs after GM-CSF and RPM induction added to T cells proliferation system for 72 h. PMA (50 ng/ml), ionomycin (750 ng/ml) and GolgiStop were added in the last 6 hrs. The percentage of IFN-γ^+^ CD8^+^ T cells is shown. (**D**) Adoptive transfer of GM-CSF-induced CD11b^+^ Ly6C^high^ cells improves graft survival while GM-CSF + RPM-induced CD11b^+^ Ly6C^high^ cells accelerated skin graft rejection in a male skin→female mouse model. Male C57BL/6 skin grafts were transplanted onto female C57BL/6 recipients (n = 10, white circles) or recipients transferred with 5 × 10^6^ CD11b^+^ Ly6C^hi^Ly6G^−^ cells induced by GM-CSF (n = 12, black circle) or GM-CSF with RPM (n = 8, triangles) at days 0 and 7. (**E**) The percentages of M-MDSCs and G-MDSCs induced from WT or Lyzs-mTOR KO BM cells by GM-CSF. (**F**) The effects of the sorted CD11b^+^ Ly6C^hi^Ly6G^−^ M-MDSCs induced from WT or Lyzs-mTOR KO BM cells on T cell proliferation were measured by CFSE dye dilution. (**G**) The percentages of M-MDSCs induced from WT or Lyzs-rictor KO BM cells by GM-CSF for 4 days. The numbers in FACS plots represent the percentages of cells in the gates. Results are expressed as mean ± SD. Three independent experiments with similar results were done. **p < 0.01, and ***p < 0.001 for comparison between the indicated groups.

In order to further gain insight into the effects of mTOR on M-MDSCs, we used a standard MDSC induction culture system^14^. As shown in Fig. 4, RPM significantly decreased the differentiation of CD11b^+^ Ly6C^high^ M-MDSCs from bone marrow myeloid progenitors (P < 0.01, Fig. 4A). These RPM-treated CD11b^+^ Ly6C^high^ monocytic cells failed to inhibit T cell proliferation and cytokine production in contrast to RPM-untreated CD11b^+^ Ly6C^high^ monocytic cells which displayed efficient immunosuppression on CD4^+^ and CD8^+^ T cell proliferation and IFN-γ expression in CD8^+^ T cells (P < 0.001, Fig. 4B,C, Suppl. Fig. 14). In consistent with the *in vitro* functional studies, the RPM-treated CD11b^+^ Ly6C^high^ M-MDSCs did not delay but even accelerated skin graft rejection in a male skin→female recipient model after adoptive transfer of the sorted CD11b^+^ Ly6C^high^ monocytic cells into syngeneic female recipients (P < 0.05, Fig. 4D), whereas the adoptive transfer of the induced CD11b^+^ Ly6C^high^ monocytic cells without RPM significantly prolonged skin graft survival in the same model (P < 0.01, Fig. 4D), supporting that the GM-CSF-induced CD11b^+^ Ly6C^high^ M-MDSCs had immunosuppressive ability to prevent graft rejection. The accelerated skin graft rejection by RPM-treated CD11b^+^ Ly6C^high^ M-MDSCs is likely due to the increased inflammatory cytokines such as IL-12 and IL-1β by these M-MDSCs (Suppl. Fig. 15). Furthermore, mTOR deletion in myeloid cells also decreased CD11b^+^ Ly6C^high^ M-MDSC differentiation (Fig. 4E) and immunosuppressive function (Fig. 4F, Suppl. Fig. 16) as determined by the percentages and the inhibiting ability on T cell proliferation of M-MDSCs derived from either mTOR KO and WT bone marrow myeloid progenitors in the *in vitro* culture systems as described in the materials and methods. These data collectively indicate that mTOR is an intrinsic factor to control M-MDSC differentiation.

mTOR, composed of two distinct complexes, mTOR complex 1 (mTORC1) and mTORC2, has been studied extensively in a variety of biological systems^30^. mTORC1 is closely involved in translation initiation, autophagy inhibition and lipid biosynthesis, whereas mTORC2 promotes actin rearrangement and uptake of nutrients^31^. It is widely known that mTORC1 is sensitive to RPM and mTORC2 is somehow resistant to RPM. However, RPM acts on both mTORC1 and mTORC2 in some cases depending on the doses and the duration of RPM treatment^32^. To see whether mTORC2 pathway is involved in the induction of M-MDSCs, we employed mice with a myeloid-specific deficiency of rictor (Lyzs-rictor KO) that is a key component of mTORC2^33^ and compared the levels of CD11b^+^ Ly6C^high^ M-MDSC subpopulation in WT mice and Lyzs-rictor KO mice after alloskin grafting. There was no significant difference for the percentages of CD11b^+^ Ly6C^high^ monocytic cells in the spleens of these mice at 7 days after grafting (Fig. 4G). The induced M-MDSCs from Lyzs-rictor KO and WT bone marrow cells have similar immunosuppressive ability on T cell proliferation and produced similar levels of NO as WT M-MDSCs of skin-grafted mice (Suppl. Fig. 17). These studies indicate that mTORC2 may not participate in the regulation on M-MDSC differentiation. Based on these *in vitro* and *in vivo* data, we conclude that RPM inhibits M-MDSC differentiation mainly through the mTORC1 pathway but not mTORC2 pathway.

### RPM blocks MDSC immunosuppressive function via iNOS pathway

**Figure 5:**
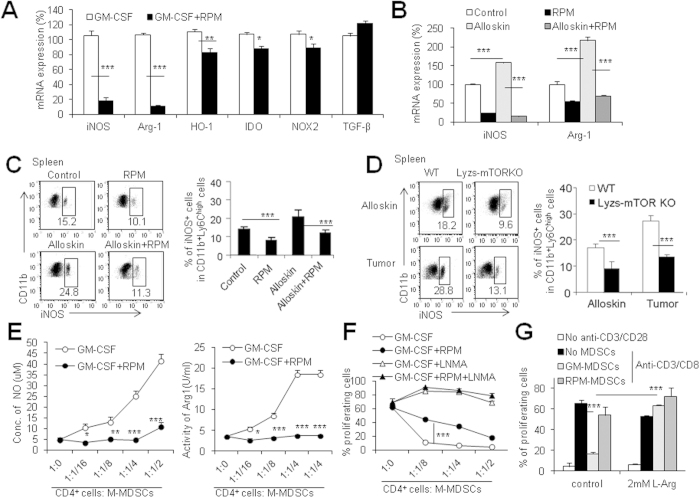
RPM blocks MDSC immunosuppressive function via iNOS pathway. (**A**) CD11b^+^ Ly6C^high^Ly6G^−^ M-MDSCs were sorted from GM-CSF-induced MDSCs with or without RPM and added into T cell proliferation system at 1:1 ratio for 72h. Then, the added CD11b^+^ cells in culture were sorted again and the mRNA expression of indicated genes were determined by real-time PCR. iNOS and Arg1 mRNA expression (**B**) and iNOS protein expression (**C**) in CD11b^+^ Ly6C^high^Ly6G^−^ M-MDSCs isolated from spleens of control mice, RPM treated mice, alloskin-grafted mice and RPM treated alloskin-grafted mice at day 7. (**D**) iNOS protein expression in CD11b^+^ Ly6C^high^Ly6G^−^ M-MDSCs in spleens of alloskin-grafted or tumor-loaded WT and lyzs-mTOR KO mice. (**E**) Nitrite concentration and arginase activity in the *in vitro* T cell suppression assays were measured as described in the materials and methods. Experiments were performed in triplicates. (**F**) 2 mM NG-monomethyl-L-arginine (L-NMMA) was added in the immunosuppression assay to see the involvement of NO in M-MDSC-mediated immunosuppression. (**G**) For addition experiments, L-arginine (2 mM) was added in the immunosuppression assays to see whether depletion of L-arginine in the medium caused by M-MDSCs is involved in the immunosuppressive process. The numbers in FACS plots represent the percentages of cells in the gates. Results are expressed as mean ± SD. Three independent experiments showing similar results were performed. **p < 0.01, and ***p < 0.001 compared between the indicated groups.

It is reported that MDSCs inhibit T cell function via multiple pathways including up-regulation of iNOS, Arg-1, HO-1, IDO, NOX2 and TGF-β^34^,^35^. We thus detected these gene expressions in GM-CSF-induced CD11b^+^ Ly6C^high^ M-MDSCs in the presence and absence of RPM by real-time PCR as described in methods. The presence of RPM dramatically inhibited iNOS and Arg-1 expressions in M-MDSCs by about 80%, whereas RPM slightly but significantly decreased HO-1, IDO, and NOX2 expressions in these cells (P < 0.05, Fig. 5A). Alloskin grafting promoted iNOS and Arg-1 mRNA expression in sorted splenic CD11b^+^ Ly6C^high^ M-MDSCs as determined by real-time PCR (Fig. 5B). The up-regulated iNOS expression in splenic CD11b^+^ Ly6C^high^ M-MDSCs of alloskin-grafted mice was also observed by intracellular staining flow cytometry assays (P < 0.001, Fig. 5C). RPM therapy almost completely blocked the alloskin grafts-promoted up-regulation of iNOS in these cells (P < 0.001, Fig. 5C). Similar to the alterations in allogeneic transplant models, The iNOS expression in the splenic CD11b^+^ Ly6C^high^ M-MDSCs of alloskin-grafted or tumor-bearing Lyzs-mTOR KO recipients were significantly decreased than WT recipients as detected by flow cytometry (P < 0.001, Fig. 5D). Furthermore, the concentrations of NO in the culture medium and the Arg activity in the CD11b^+^ Ly6C^high^ M-MDSCs were detected after co-culture of the induced M-MDSCs with T cells as performed for the immunosuppression assays. These results showed that RPM pre-treatment significantly blocked the iNOS and Arg activities in GM-CSF-induced CD11b^+^ Ly6C^high^ M-MDSCs in the co-culture system (P < 0.001, Fig. 5E).

To determine whether NO production is essential for GM-CSF-induced CD11b^+^ Ly6C^high^ M-MDSCs to mediate its immunosuppressive function, we blocked NO production by adding a specific iNOS inhibitor, L-NMMA (2 mM as reported previously^36^) into the *in vitro* MDSC function assay system as described in the materials and methods section. 2 mM L-NMMA almost completely blocked NO production by M-MDSCs in the culture system as evidenced by NO levels in the culture medium (Suppl. Fig. 18). L-NMMA efficiently blocked the immunosuppressive effects of CD11b^+^ Ly6C^high^ M-MDSCs on T cell proliferation (P < 0.001, Fig. 5F). NO generation is catalyzed by inducible nitric oxide synthase (iNOS) converting arginine into citrulline and NO. However, Arg inhibitor fails to show the remarkable blockage on the inhibitory effect on T cell proliferation of CD11b^+^ Ly6C^high^ M-MDSCs, although a slightly reversed effect of Arg inhibitor on the immunosuppressive function of M-MDSCs were detected in certain conditions (Suppl. Fig. 19). To see whether arginine depletion of the culture medium caused by increased iNOS and Arg-1 activities of M-MDSCs contributes to the immunosuppressive effect of CD11b^+^ Ly6C^high^ M-MDSCs on T cell responses, we added more L-arginine (2 mM) to the culture system. Additional supplement of arginine significantly reversed the inhibiting effects of CD11b^+^ Ly6C^high^ M-MDSCs on T cell proliferation (P < 0.001, Fig. 5G), indicating that the possible depleting arginine caused by increased iNOS/Arg1 activities in CD11b^+^ Ly6C^high^ M-MDSCs is likely to be the key reason for the immunosuppression of M-MDSCs induced in the present system. Thus, these data support the conclusion that inhibition on iNOS and Arg-1 by RPM or mTOR deletion is a key pathway for the poor immunosuppressive ability of RPM-treated CD11b^+^ Ly6C^high^ M-MDSCs.

### mTOR controls M-MDSC differentiation through mastering cellular metabolism

**Figure 6:**
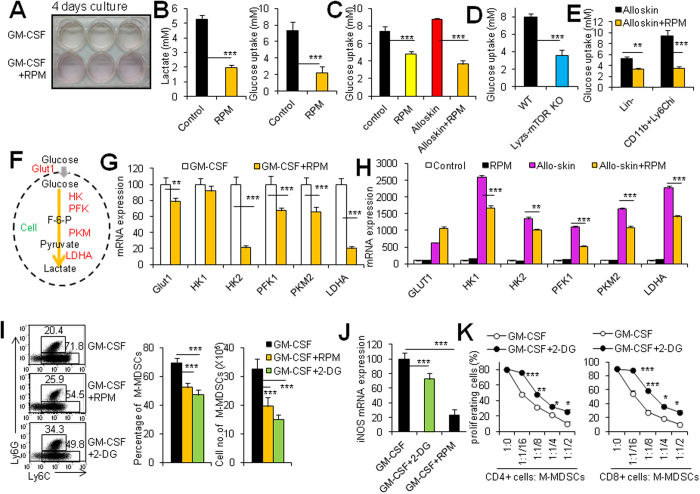
mTOR controls M-MDSC differentiation through mastering cellular metabolism. MDSCs induced from bone marrow cells after 4 days of culture in the presence of 40 ng/ml recombinant mouse GM-CSF with or without 1 μM RPM. (**A**) The macroscopic pictures of cell culture. (**B**) Lactate production and glucose uptake of M-MDSCs. (**C**) Glucose uptake of M-MDSCs isolated from spleens of control mice, RPM-treated mice, alloskin-grafted mice and RPM-treated alloskin-grafted mice at day 7 post transplantation. (**D**) Glucose uptake of M-MDSCs isolated from spleens of alloskin-grafted WT and lyzs-mTOR KO mice at day 7 after skin grafting. (**E**) Glucose uptake of Lin^−^ cells isolated from bone marrows of alloskin-grafted mice and RPM-treated alloskin-grafted mice at day 7 after skin grafting. (**F**) Schematic representation of the important genes (red) involved in the glycolysis pathway. (**G**) Some key genes involved in the glycolysis were detected in GM-CSF-induced M-MDSCs (white bars) and GM-CSF + RPM-induced M-MDSCs (yellow bars) by real-time PCR. (**H**) Some genes involved in the glycolysis process were detected in M-MDSCs isolated from spleens of control mice, RPM-treated mice, alloskin-grafted mice and RPM-treated alloskin-grafted mice at day 7 after skin grafting. MDSCs were induced from bone marrow cells after 4 days of culture in the presence of 40 ng/ml recombinant mouse GM-CSF, GM-CSF + 1 μM RPM or GM-CSF + 1.5 mM 2-DG. (**I**) Percentages and cell numbers of CD11b^+^ Ly6C^high^Ly6G^−^ M-MDSCs in these three groups were analyzed. The numbers in FACS plots represent the percentages of cells in the gates. (**J**) iNOS mRNA expressions of CD11b^+^ Ly6C^high^Ly6G^−^ M-MDSCs were determined by real-time PCR. (**K**) Sorted CD11b^+^ Ly6C^high^Ly6G^−^ M-MDSCs from different induction groups were assayed for their immunosuppressive ability. Results are expressed as mean ± SD (N = 4). More than two independent experiments showing similar results were done. **p < 0.01, and ***p < 0.001 for comparison between the indicated groups.

In order to understand why RPM decreases M-MDSC differentiation from its precursors, we detected the cell proliferation and cell death ratio during MDSC induction *in vitro*. RPM treatment did not remarkably alter cell proliferation and cell death of CD11b^+^ Ly6C^high^ cells during M-MDSC induction (Suppl. Fig. 20), excluding the possibility that the changed cell proliferation and death contribute to the poor M-MDSC differentiation caused by RPM treatment. However, it causes our great caution that the culture medium of bone marrow MDSCs induction system is susceptible to change the color to be yellow and the presence of RPM obviously blocked this process (Fig. 6A), indicating that high glycolysis may occur during MDSC differentiation from bone marrow cells *in vitro*. This speculation is confirmed by the biochemical assays showing that RPM treatment significantly decreased glucose uptake and lactate production during MDSC induction *in vitro* (P < 0.001, Fig. 6B). RPM or mTOR deletion also decreased glucose uptake of splenic CD11b^+^ Ly6C^high^ M-MDSCs isolated from alloskin-grafted mice (P < 0.001, Fig. 6C,D). Importantly, bone marrow Lin^−^ cells and CD11b^+^ Ly6C^high^ cells displayed a decreased glucose uptake when alloskin-grafted mice were treated with RPM compared with these cells of RPM-untreated alloskin-grafted mice (P < 0.01, Fig. 6E). In addition to glucose transport 1 (Glut1) that serves as plasma membrane transporters for glucose uptake, a panel of key enzymes are involved in the spectrum of the glycolytic pathway for cellular glucose utilization to generate lactate and ATP molecules (Fig. 6F). Real-time PCR analyses revealed that RPM markedly down-regulated these genes encoding glycolysis-related molecules, including the transporter Glut1 and glycolytic enzymes hexokinase 2 (HK2), phosphofructokinase 1 (PFK1), pyruvate kinase muscle (PKM), and LDHA (lactate dehydrogenase-α) in the sorted GM-CSF-induced CD11b^+^ Ly6C^high^ M-MDSCs (Fig. 6G). In agreement with the *in vitro* results, RPM treatment also significantly decreased HK, PFK1, PKM, and LDHA gene expression in CD11b^+^ Ly6C^high^ M-MDSCs in alloskin-grafted mice (Fig. 6H). These data collectively indicate that RPM significantly blocks the glycolytic pathway during M-MDSC induction *in vitro* and *in vivo*.

To directly test the importance of the metabolic reprogramming in M-MDSC differentiation, we induced M-MDSC differentiation from bone marrow cells in the presence or absence of 2-DG, a prototypical inhibitor of the glycolytic pathway via blocking hexokinase, the first rate-limiting enzyme of glycolysis. A low dose of 2-DG treatment results in a significantly decreased differentiation of CD11b^+^ Ly6C^high^ M-MDSCs as RPM does (P < 0.001, Fig. 6I). In parallel, the iNOS expression and the inhibitory ability of CD11b^+^ Ly6C^high^ M-MDSCs are also decreased when 2-DG exists during M-MDSC differentiation (P < 0.01, Fig. 6J,K). These data indicate that glycolytic pathway is required for M-MDSC differentiation from bone marrow precursors.

**Figure 7:**
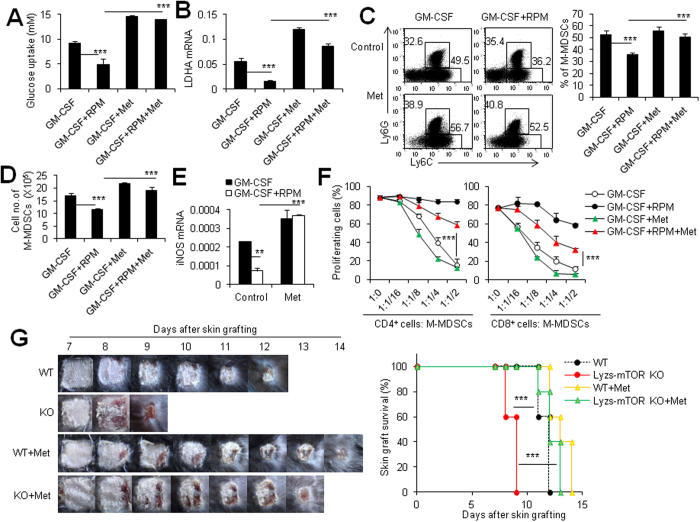
Enhancement of glycolysis by metformin partially rescues the phenotype and function of mTOR-deficient M-MDSCs. MDSCs induced from bone marrow cells after 4 days of culture in the presence of 40 ng/ml recombinant mouse GM-CSF or GM-CSF + RPM(1 μM) or GM-CSF + metformin(2 mM) or GM-CSF + RPM(1 μM) + metformin(2 mM). Glucose uptake (**A**) and LDHA mRNA expression (**B**) of M-MDSCs from these four induction groups were determined. The percentages (**C**), and the cell numbers (**D**) of M-MDSCs from the four induction groups. The numbers in FACS plots represent the percentages of cells in the gates. (**E**) Metformin rescues the RPM-decreased iNOS mRNA expression in M-MDSCs. (**F**) Metformin significantly rescues the RPM-decreased immunosuppression on T cells of M-MDSCs. Results are expressed as mean ± SD of triplicates. Three independent experiments with identical results were done. **p < 0.01, and ***p < 0.001 for comparison between the indicated groups. (**G**) BALB/c tail skin were transplanted onto WT or lyzs-mTOR KO mice and recipients treated with Metformin at dose of 150 mg/kg from day 0 to day 7 after skin grafting. Macroscopic pictures of alloskin grafts were collected at different time points (left panel). Graft survival rates were compared by the log-rank test (right panel). Six to 8 mice in each group were performed. **p < 0.01, and ***p < 0.001 compared between the indicated groups.

Metformin is widely used for treating diabetes mellitus for many years^37^,^38^. In contrast to 2-DG, metformin increases glucose uptake and glycolysis through 5′-AMP-activated protein kinase (AMPK)-dependent and independent pathways^39^,^40^,^41^,^42^. Thus, we asked whether metformin impacts on M-MDSCs in an opposite way as 2-DG and can rescue the inhibitory action of RPM on M-MDSCs. As shown in Fig. 7, metformin significantly increased glucose uptake and LDHA expression in GM-CSF-induced CD11b^+^ Ly6C^high^ M-MDSCs (P < 0.01, Fig. 7A). It also significantly reversed the RPM-reduced glucose uptake and LDHA expression during M-MDSC induction (P < 0.001, Fig. 7A,B). Importantly, metformin treatment almost completely rescued the inhibitory effect of RPM on the differentiation of CD11b^+^ Ly6C^high^ M-MDSCs as evidenced by the recovered percentages (P < 0.01, Fig. 7C), and cell numbers (P < 0.01, Fig. 7D) of CD11b^+^ Ly6C^high^ M-MDSCs. In addition, metformin also improved the iNOS expression (P < 0.001, Fig. 7E) and partially the immunosuppressive function *in vitro* (P < 0.01, Fig. 7F) of RPM-treated M-MDSCs. Furthermore, metformin treatment significantly delayed alloskin graft rejection in Lyzs-mTOR KO recipients (Fig. 7G). Thus, all these data collectively support that mTOR-dependent glycolytic pathway is closely required for the differentiation of CD11b^+^ Ly6C^high^ M-MDSCs from bone marrow precursors.

## Discussion

Our present data demonstrate that RPM significantly inhibits M-MDSC differentiation and immunosuppressive function in recipients with allo-grafts or tumors. mTOR-deficiency in myeloid cells causes a significantly accelerated rejections of alloskin and tumor grafts in mice, due to the decreased cell number and the defect immunosuppressive ability of CD11b^+^ Ly6C^high^ M-MDSCs. RPM blocks CD11b^+^ Ly6C^high^ M-MDSC differentiation from its precursors through inhibiting glycolysis. Thus, we conclude that mTOR is an intrinsic regulator for the differentiation and function of immunosuppressive CD11b^+^ Ly6C^high^ M-MDSCs in mice transplanted with allo-grafts and tumors through controlling cellular metabolism. The decreased CD11b^+^ Ly6C^high^ M-MDSCs may contribute to the clinical anti-tumor action of RPM treatment, which offers new cellular mechanisms for RPM to treat cancers in addition to its direct inhibitory effects on tumor cells. On the other hand, the inhibitory effects of RPM on M-MDSCs may potentially block M-MDSC-mediated immune tolerance induction in allogeneic transplant recipients.

RPM, a specific mTOR inhibitor, is widely used in clinics to prevent allograft rejection. However, the usage of RPM in kidney transplantation is challenging due to the frequent occurrence of various side effects including inflammatory manifestations such as pneumonitis, glomerulonephritis or chronic inflammatory anaemia^22^. Proteinuria, which is another frequently observed side effect in patients treated with mTOR inhibitors, seems to originate from the activation of the innate immune system^43^. The pro-inflammatory effects of RPM are recently supported by the immunological analysis of kidney transplant patients as well as in experimental animal models^24^,^25^,^44^,^45^. In the present study, we find that RPM or mTOR deletion causes the deficiency of immunosuppressive CD11b^+^ Ly6C^high^ M-MDSCs in transplant and tumor-bearing mouse models. The involvement of MDSCs in transplantation and tumors has well been demonstrated in various models^2^,^46^,^47^. Our results show that mTOR deficiency in myeloid cells by either immunosuppressant drug RPM or genetic deletion significantly decreased both CD11b^+^ Ly6C^high^ M-MDSC cell numbers and the later inhibitory effects on the immunity against allo-grafts and tumors. The poor immunosuppressive ability and the enhanced inflammatory function of CD11b^+^ Ly6C^high^ M-MDSCs likely contribute to the accelerated alloskin and tumor graft rejection in mTOR KO recipients. Blocking iNOS pathways markedly reversed the immunosuppressive effect of CD11b^+^ Ly6C^high^ M-MDSCs on T cell response, indicating that iNOS pathway is one of the major pathways for CD11b^+^ Ly6C^high^ M-MDSCs to process immunosuppression in these models as reported previously^14^. In contrast, mTOR-deficient CD11b^+^ Ly6C^high^ M-MDSCs expressed low level of iNOS and Arg-1, which subsequently cause the poor immunosuppression of M-MDSCs. Therefore, RPM down-regulates host immunity via directly inhibiting T cell immune response and simultaneously promotes host inflammatory response by inhibiting immunosuppressive M-MDSCs.

mTOR is evolutionary conserved signaling molecules modulating the sensing of cellular metabolic state and directing the cell functional fate^48^. mTOR forms two distinct multi-protein complexes, mTORC1 and mTORC2. mTORC1 is regarded as a master regulator of cell growth and metabolism^49^. Recent evidence supports an important cross-talk between immune system and cellular metabolic regulation, bringing into the spotlight an unexpected layer of metabolic regulation of immune system. Activation of the inflammatory response is accompanied by a metabolic shift to aerobic glycolysis^50^,^51^. Although aerobic glycolysis produces less ATP per glucose molecule compare to tricarboxylic acid cycle, less enzymatic steps are involved so that it can be easier enhanced and more ATP could be produced in a short time. Thus, aerobic glycolysis is very important to meet energy needs for activated immune cells^52^. Besides regulating immune cell function, glycolysis also regulates certain immune cell lineage development, such as mTORC1-dependent metabolic pathways program DC lineage development^53^. We herein found that mTORC1-dependent glycolysis is critical for the lineage commitment of CD11b^+^ Ly6C^high^M-MDSCs but not CD11b^+^ Ly6C^medium^ G-MDSCs. This conclusion is supported by the following observations: 1) mTOR inhibition or deletion significantly decreased glycolysis during MDSC differentiation as evidenced by the lower glucose uptake and glycolysis-related enzymes like HK1, HK2, PFK1, PKM2 and LDHA. 2) 2-DG, a prototypical inhibitor of the glycolytic pathway through blocking hexokinase, significantly inhibits CD11b^+^ Ly6C^high^ M-MDSC development from bone marrow as RPM does. 3) Metformin, an enhancer of the glycolytic pathway, significantly enhances CD11b^+^ Ly6C^high^ M-MDSC differentiation and remarkably rescues RPM-mediated poor differentiation of MDSCs *in vitro* and *in vivo*. On the other hand, mTORC2 deficiency failed to cause significant impacts on CD11b^+^ Ly6C^high^ M-MDSCs in respect of differentiation and function. Therefore, RPM inhibits M-MDSC differentiation from bone marrow precursors mainly through mTORC1-dependent glycolysis pathway.

Metformin has become a mainstay in the modest therapeutic armamentarium for the treatment of the insulin resistance of type 2 diabetes mellitus^54^. The primary cellular target for metformin is complex I of mitochondrial oxidative phosphorylation and leads to a reduction in energy charge. A decrease in energy charge significantly activates glycolysis by releasing ATP-mediated inhibition of L-type pyruvate kinase and phosphofructokinase^55^,^56^. In our studies, we observed that the alloskin graft rejection was significantly delayed when Lyzs-mTOR KO recipients were treated with metformin. This result suggested that the glycolysis of innate immune cells was very important for graft rejection. Additionally, metformin significantly increased the production and function of CD11b^+^ Ly6C^high^ M-MDSCs during M-MDSC induction *in vitro*. This result is in accordance with a recent report showing that metformin attenuates PMA-induced monocyte-to-macrophage differentiation and pro-inflammatory cytokines production^57^. These results indicate that increasing glycolysis may inhibit monocyte maturation and increase immature CD11b^+^ Ly6C^high^ M-MDSC development. It is speculated that the increased M-MDSCs by metformin treatment in diabetes patients may be beneficial for the host to prevent the pathogenesis of inflammatory complicates in addition to controlling blood sugar levels.

In the present study, we used Lysz-Cre strategy to delete mTOR in monocytes/macrophages. mTOR was efficiently deleted in monocytes/macrophages and granulocytes as determined by real-time PCR, western blots and mTOR activity indicated by p-S6. On the other hand, Lyzs was mainly expressed in CD11b^+^ Ly6C^high/medium^ cells, whereas almost no CD11b^+^ CD11c^+^ dendritic cells express Lyzs, as determined by Lyzs-GFP reporter mice. However, it should be pointed out that Lyzs-Cre may not be expressed in an identical manner to Lyzs-GFP reporter. The deletion of mTOR in CD11c^+^ dendritic cells in Lysz-mTOR KO mice needs to be further clarified.

In summary, mTORC1 intrinsically controls the immunosuppressive CD11b^+^ Ly6C^high^ M-MDSC maturation and function by mediating cellular glycolysis activity. RPM and 2-DG treatments significantly decrease CD11b^+^ Ly6C^high^ M-MDSCs respectively by blocking glycolysis; in contrast, metformin significantly increases CD11b^+^ Ly6C^high^ M-MDSCs by enhancing glycolysis (Suppl. Fig. 21). Additional insights into the control of MDSC differentiation by cellular metabolism will facilitate the application of MDSCs to treat immune disorders and help us to use these cellular metabolism-relevant agents to modify host inflammation in purpose.

## Materials and Methods

### Mice

BALB/c, C57BL/6 (B6) were purchased from the Beijing Laboratory Animal Research Center (Beijing, China). The myeloid -specific mTOR conditional knockout mice (LyzsCre-mTOR^loxp/loxp^) and myeloid cell-specific rictor conditional knockout mice (LyzsCre-rictor^loxp/loxp^) under C57BL/6 genetic background were obtained by crossing mTOR^loxp/loxp^ mice and rictor^loxp/loxp^ mice with mice expressing Cre recombinase under the control of the Lysozyme promoter (Lyzs-Cre) respectively^25^,^33^. Lyzs-Cre-negative, mTOR^loxp/loxp^ littermates served as the wild-type (WT) control. Six-to-8-week-old mice (male and female) were usually used for the *in vitro* experiments. mTOR^loxp/loxp^ mice were kindly provided by Dr Zhongzhou Yang from Center of Model Animal Research at Nanjing University, China. Lyzs-Cre mice were kindly offered by Dr. Lianfeng Zhang, Key Laboratory of Human Diseases Comparative Medicine, Ministry of Health; Institute of Laboratory Animal Science, CAMS & PUMC. Primers used to identify genetically modified mice are described previously^25^,^33^. All animal experiments were performed in accordance with the institutional guidelines and approval of the Animal Ethics Committee of the Institute of Zoology, Beijing, China.

### Mouse model of acute alloskin graft rejection

For acute alloskin rejection model, tail skin from BALB/c mice were transplanted into recipients and the judgment of the end point of rejection as described previously^14^. Photographs were taken daily with a digital camera (Canon Powershot A640; Canon, Tokyo, Japan) until the graft was rejected completely.

### Chronic skin graft rejection mouse model and adoptive transfer of MDSCs

Adoptive transfer of MDSCs in the chronic skin rejection mouse model was performed as reported previously^58^. Briefly, male C57BL/6 skin grafts were transplanted onto untreated female C57BL/6 recipients. In some cases, recipients were i.v transferred with 5 × 10^6^ sorted CD11b^+^ Ly6C^high^Ly6G^−^ M-MDSCs which derived from C57BL/6 bone marrow cells in the presence of GM-CSF and/or RPM at day 0 and day 7. Graft survival rates were compared by the log-rank test.

### Tumor-bearing mouse model

WT and lyzs-mTOR KO mice with the same age and sex were injected s.c. with 1.5 × 10^6^ EL4 lymphoma. The tumor size was measured from day 5 to day 15, when tumors reached a maximum.

### Drug treatment

C57BL/6 recipients were transplanted with alloskin grafts and intraperitoneally administered with RPM at a dose of 1.5 mg/kg from day −3 to day 7^19^. WT and Lyzs-mTOR KO mice were transplanted with alloskin grafts and intraperitoneally administered with Metformin (PHR1084, sigma) at a dose of 150 mg/kg from day 0 to day 7.

### Detection of anti-donor Abs in the serum

The anti-donor alloreactive Abs in the sera of BALB/c skin-grafted C57BL/6 mice were measured by flow cytometry. Briefly, 5 × 10^5^ BALB/c splenocytes were used as target cells and incubated for 30 min at 4 °C with either negative control serum (unimmunized mice, diluted 1:10) or with the recipient’s sera (diluted 1:10). Then, the cells were washed three times and incubated with optimal concentrations of FITC-conjugated goat anti-mouse IgG and IgG2b Abs (Santa Cruz Biotechnology) respectively for 30 min at 4 °C in the dark. Samples were assayed by flow cytometry. Results were expressed as the mean fluorescence intensity of stained cells subtracted by the mean fluorescence intensity of cells incubated with negative control serum and FITC-labeled secondary Abs.

### Isolation and induction of MDSCs

CD11b^+^ Ly6C^high^ (M-MDSCs) and CD11b^+^ Ly6C^medium^ (G-MDSCs) cells were isolated from spleens and bone marrow of alloskin-transplanted mice or tumor-bearing mice using cell sorter of MoFlo XDP (Beckman Coulter). The purity of cell populations was >99%. For *in vitro* induction, bone marrow cells were prepared as described previously^14^. The method of MDSC induction from bone marrow cells is done according to the paper reported previously^58^. Briefly, 4 × 10^6^ bone marrow cells per well were cultured in a 6-well plate, in 2 ml of medium for each well supplemented with 40 ng/ml mouse recombinant GM-CSF and 1uM RPM (Sigma-Aldrich, dissolved in DMSO). For the experiments with metformin or 2-deoxyglucose (2-DG, Sigma-Aldrich), 2 mM metformin or 1.5 mM 2-DG was added in culture at day 0. Cells were maintained at 37 °C, 5% CO_2_ for 4 days and then two population-CD11b^+^ Ly6C^high^ (M-MDSCs) and CD11b^+^ Ly6C^medium^ (G-MDSCs) cells are sorted for immunosuppressive functional assay. Cell culture medium was DMEM supplemented with 2 mM L-glutamine, 20 μM 2-ME, penicillin (100 U/ml), streptomycin (0.1 mg/ml), and 10% heat inactivated FBS.

### Assays for the suppression on T cells of M-MDSCs

Spleen cells from C57BL/6 mice were labeled with CFSE (Invitrogen) according to the manufacturer’s instructions. 2 × 10^5^/well CFSE-labeled spleen cells were stimulated with coated 1μg/ml anti-CD3 and 5 μg/ml soluble anti-CD28 mAbs for 3 days and co-cultured at the indicated ratios with sorted M-MDSCs or G-MDSCs in 96 flat bottom well plates^14^. After 3 days, cells were stained with CD4 and CD8 (BD pharmingen), and CFSE signal of gated CD4^+^ and CD8^+^ lymphocytes was analyzed respectively. The extent of cell proliferation was quantified by ModFit LT software V3.0 (Verity Software House, Inc.) The culture medium consisted of RPMI 1640 medium supplemented with L-glutamine (2 mM), penicillin (100 U/ml), streptomycin (0.1 mg/ml), 2-ME (50 μM), and 10% heat-inactivated FBS. For experiments which examined the effect of NO and arginase, 2 mM NG-monomethyl-L-arginine (L-NMMA; Sigma-Aldrich) or 1 mM nor-NOHA (Calbiochem, San Diego, CA) were added at the beginning of the culture as reported previously^14^. For L-arginine addition experiment, we use custom-formulated Advanced RPMI 1640 (GIBCO, Invitrogen) with L-arginine at 150 μM and L-arginine (2 mM) was added to the culture system.

### Isolation of infiltrated cells in alloskin grafts and transplanted tumors

Skin grafts at day 7 and tumor at day 10 were retrieved and minced into small pieces with a scalpel and then digested for 1 h at 37 °C in RPMI 1640 medium containing 30 U/ml collagenase (type IV; Sigma-Aldrich). Collagenase pretreated tissues were then ground with the plunger of a 5-ml disposable syringe and passed through a 70 μm nylon cell strainer. Cells were collected after centrifugation at 300 × g for 10 min and re-suspended in FACS staining buffer for cell surface marker staining as described below.

### Cell isolation and flow cytometry analysis

Bone marrow cells, splenocytes, peripheral white blood cells and draining lymph node cells were prepared as described previously^59^. For isolation of infiltrated cells in alloskin grafts and transplanted tumor, skin grafts at day 7 and tumor at day 10 were retrieved and minced into small pieces with a scalpel and then digested for 1 h at 37 °C in RPMI 1640 medium containing 30 U/ml collagenase (type IV; Sigma-Aldrich). Collagenase pretreated tissues were then ground with the plunger of a 5-ml disposable syringe and passed through a 70 μm nylon cell strainer. Cells were collected after centrifugation at 300 × g for 10 min and re-suspended in FACS staining buffer for cell surface marker staining. Cells were stained with optimized Ab dilutions. For surface marker staining, the following Ab–fluorochrome combinations were used: PE-Cy5–anti-mCD11b (M1/70), PE or PE-Cy5–anti-mGr1 (RB6-8C5), PE or FITC–anti-mLy6C (AL-21), PE–anti-mLy6G (1A8), PE-Cy5–anti-mCD4 (RM4-5), PE or FITC–anti-mCD8a (53-6.7). For Intracellular iNOS and cytokine staining, FITC–anti-miNOS (6/iNOS/NOS TypeII), PE–anti-mIFN-γ (XMG1.2) mAb and PE–anti-mIL-2 (JES6-5H4) mAb were used and the method was previously described^14^,^60^. Abs were purchased from eBioscience, BioLegend, or BD Pharmingen. Samples were analyzed on a Beckman Coulter Epics XL benchtop FCM (Beckman Coulter) with FlowJo (Tree Star, San Carlos, CA) software.

### Quantitative PCR analysis

Total RNA was isolated with TRIzol (Invitrogen, Carlsbad, CA) and contaminating DNA was removed by on-column treatment with RNase-free DNase (Qiagen). Reverse transcription was performed with M-MLV superscript reverse transcriptase according to the manufacturer’s instructions^61^. Real-time PCR was performed using multiple kits (SYBR Premix Ex TaqTM, DRR041A, Takara Bio) on CFX96 (Bio-Rad). The primers used in the present study are listed in Table 1. The mRNA expression levels of each gene were normalized to the expression level of the housekeeping gene hypoxanthine phosphoribosyl transferase.

### Detection of NO in cell culture medium

After incubating equal volumes of culture supernatants or serum (100 μl) with Greiss reagent (1% sulfanilamide in 5% phosphoric acid and 0.1% N-1-naphthylethylenediamine dihydrochloride in double-distilled water) at room temperature for 10 min, the absorbance at 550 nm was measured using a microplate reader (Bio-Rad)^14^. Nitrite concentrations were determined by comparing the absorbance values for the test samples to a standard curve generated by serial dilution of 0.25 mM sodium nitrite.

### Arginase activity assay

Arginase assay was described previously^25^. Briefly, 10^6^ cells were mixed with 100 μl of 0.1% Triton X-100. After 30 mins of incubation on a shaker, 100 μl of 25 mM Tris-HCl and 20 μl of 10 mM MnCl_2_ were added to the sample. The arginase was then activated by heating the sample for 10 mins at 56 °C, and arginine hydrolysis was conducted by incubating the sample with 100 μl of 0.5 M L-arginine (pH 9.7) at 37 °C for 60–120 mins. The reaction was stopped with 900 μl of H_2_SO_4_ (96%)/H_3_PO_4_ (85%)/H_2_O, and the sample was mixed with 40 μl of 9% isonitrosopropiophenone. Absorbance was read at 540nm in a microplate reader. All samples were read in triplicate.

### Glucose uptake assay

The glucose uptake by M-MDSCs was determined by glucose uptake fluorometric assay kit (MAK084, Sigma-Aldrich). Briefly, sorted M-MDSCs were seeded at 1 × 10^5^ cells/well in a 96 well plate, and starved in 100 μl of serum-free medium for over 4 hrs. Cells were then washed 3 times with PBS and then glucose-starved by plating with 100 μl of KRPH buffer containing 2% BSA for 40 minutes. Cells were then stimulated with or without insulin (1 μM) for 20 mins. Add 10 μl of 10 mM 2-DG, mix, and incubate for 20 mins. Following incubation, wash cells 3 times with PBS. Lyse cells with 80 μl of Extraction buffer. Freeze/thaw cells in dry ice/ethanol bath or liquid nitrogen, and then heat at 85 °C for 40 minutes. Cool cell lysate on ice for 5 mins and then neutralize by adding 10 μl of neutralization buffer. Briefly spin down at 13,000 g to remove insoluble material. Mixed 50μl supernatant with 50ul of the Master Reaction Mix to each of the wells and incubate the reaction for 40 minutes at 37 °C. Fluroscence intensity was measured at λ ex = 535/λ em = 587 nm. All readings subtracted the negative control and the amount of accumulated 2-DG6P present in the samples can be determined from the standard curve.

### Lactate production assay

Cell culture medium was collected at end point of MDSC induction system *in vitro*. The lactate concentrations within the medium were determined by Lactate Assay kit (MAK064, Sigma-Aldrich). Briefly, the cell culture supernatant is deproteinized with a 10kDa MWCO spin filter to remove lactate dehydrogenase. Mix 50μl supernatant with 50μl Master Reaction and incubate the reaction for 30 mins at room temperature in dark. Fluorescence intensity was detected at λ ex = 535/λ em = 587 nm. All readings subtracted the negative control and the amount of lactate present in the samples can be determined from the standard curve.

### Statistical analysis

Skin graft survival curves were calculated by the Kaplan–Meier method, using the GraphPad Prism software (GraphPad Software, San Diego, CA). The log-rank test was used for graft survival comparison. The median graft survival time (MST) is the time at which half of the subjects reached the respective score. All data are presented as the mean ± SD. Two-way ANOVA analysis was used for comparison among multiple groups with SPSS 16.0 software. Student’s unpaired t-test for comparison of means was used to compare between two groups. A P value less than 0.05 was considered to be statistically significant.

## Additional Information

**How to cite this article**: Wu, T. *et al.* mTOR masters monocytic myeloid-derived suppressor cells in mice with allografts or tumors. *Sci. Rep.* **6**, 20250; doi: 10.1038/srep20250 (2016).

## Supplementary Material

Supplementary Information

## Figures and Tables

**Table 1:** Primers used in the present study.

Genes		primer sequence (5′-3′)
iNOS	Forward primer	CACCAAGCTGAACTTGAGCG
	Reverse primer	CGTGGCTTTGGGCTCCTC
Arg1	Forward primer	CCAGAAGAATGGAAGAGTCAGTGT
	Reverse primer	GCAGATATGCAGGGAGTCACC
HO-1	Forward primer	AGGTACACATCCAAGCCGAGA
	Reverse primer	CATCACCAGCTTAAAGCCTTCT
IDO1	Forward primer	GCTTTGCTCTACCACATCCAC
	Reverse primer	CAGGCGCTGTAACCTGTGT
NOX2	Forward primer	TGTGGTTGGGGCTGAATGTC
	Reverse primer	CTGAGAAAGGAGAGCAGATTTCG
TGF-β1	Forward primer	GCTAATGGTGGACCGCAACAAC
	Reverse primer	GCACTGCTTCCCGAATGTCTG
Glut1	Forward primer	CAGTTCGGCTATAACACTGGTG
	Reverse primer	GCCCCCGACAGAGAAGATG
HK1	Forward primer	AGGGCGCATTACTCCAGAG
	Reverse primer	CCCTGTGGGTGTCTTGTGTG
HK2	Forward primer	TGATCGCCTGCTTATTCACGG
	Reverse primer	AACCGCCTAGAAATCTCCAGA
PFK1	Forward primer	TGTGGTCCGAGTTGGTATCTT
	Reverse primer	GCACTTCCAATCACTGTGCC
PKM2	Forward primer	TCGAGGAACTCCGCCGCCTG
	Reverse primer	CCACGGCACCCACGGCGGCA
LDHA	Forward primer	TGTCTCCAGCAAAGACTACTGT
	Reverse primer	GACTGTACTTGACAATGTTGGGA
HPRT	Forward primer	AGTACAGCCCCAAAATGGTTAAG
	Reverse primer	CTTAGGCTTTGTATTTGGCTTTTC

## References

[b1] Movahedi K. *et al.* Identification of discrete tumor-induced myeloid-derived suppressor cell subpopulations with distinct T cell-suppressive activity. Blood 111, 4233–4244 (2008). 18272812 10.1182/blood-2007-07-099226

[b2] Lees J. R., Azimzadeh A. M. & Bromberg J. S. Myeloid derived suppressor cells in transplantation. Curr Opin Immunol 23, 692–697 (2011). 21802931 10.1016/j.coi.2011.07.004

[b3] Wu T., Zhao Y. & Zhao Y. The roles of myeloid-derived suppressor cells in transplantation. Expert review of clinical immunology 10, 1385–1394 (2014). 25119260 10.1586/1744666X.2014.948424

[b4] Almand B. *et al.* Increased production of immature myeloid cells in cancer patients: a mechanism of immunosuppression in cancer. J Immunol 166, 678–689 (2001). 11123353 10.4049/jimmunol.166.1.678

[b5] Mandruzzato S. *et al.* IL4Ralpha + myeloid-derived suppressor cell expansion in cancer patients. J Immunol 182, 6562–6568 (2009). 19414811 10.4049/jimmunol.0803831

[b6] Youn J. I., Nagaraj S., Collazo M. & Gabrilovich D. I. Subsets of myeloid-derived suppressor cells in tumor-bearing mice. J Immunol 181, 5791–5802 (2008). 18832739 10.4049/jimmunol.181.8.5791 PMC2575748

[b7] Gabrilovich D. I. & Nagaraj S. Myeloid-derived suppressor cells as regulators of the immune system. Nat Rev Immunol 9, 162–174 (2009). 19197294 10.1038/nri2506 PMC2828349

[b8] Nagaraj S. *et al.* Altered recognition of antigen is a mechanism of CD8 + T cell tolerance in cancer. Nature medicine 13, 828–835 (2007). 10.1038/nm1609 PMC2135607 17603493

[b9] De Wilde V. *et al.* Endotoxin-induced myeloid-derived suppressor cells inhibit alloimmune responses via heme oxygenase-1. American Journal of Transplantation 9, 2034–2047 (2009). 19681826 10.1111/j.1600-6143.2009.02757.x

[b10] Obermajer N., Muthuswamy R., Lesnock J., Edwards R. P. & Kalinski P. Positive feedback between PGE2 and COX2 redirects the differentiation of human dendritic cells toward stable myeloid-derived suppressor cells. Blood 118, 5498–5505 (2011). 21972293 10.1182/blood-2011-07-365825 PMC3217352

[b11] Li H., Han Y., Guo Q., Zhang M. & Cao X. Cancer-expanded myeloid-derived suppressor cells induce anergy of NK cells through membrane-bound TGF-beta 1. J Immunol 182, 240–249 (2009). 19109155 10.4049/jimmunol.182.1.240

[b12] Baban B. *et al.* Physiologic control of IDO competence in splenic dendritic cells. J Immunol 187, 2329–2335 (2011). 21813777 10.4049/jimmunol.1100276 PMC3556270

[b13] Condamine T. & Gabrilovich D. I. Molecular mechanisms regulating myeloid-derived suppressor cell differentiation and function. Trends Immunol 32, 19–25 (2011). 21067974 10.1016/j.it.2010.10.002 PMC3053028

[b14] Wu T. *et al.* Smad3-deficient CD11b(+)Gr1(+) myeloid-derived suppressor cells prevent allograft rejection via the nitric oxide pathway. J Immunol 189, 4989–5000 (2012). 23045614 10.4049/jimmunol.1200068

[b15] Lim W. H. *et al.* A systematic review of conversion from calcineurin inhibitor to mammalian target of rapamycin inhibitors for maintenance immunosuppression in kidney transplant recipients. American journal of transplantation: official journal of the American Society of Transplantation and the American Society of Transplant Surgeons 14, 2106–2119 (2014). 10.1111/ajt.12795 25088685

[b16] Beck J. T., Ismail A. & Tolomeo C. Targeting the phosphatidylinositol 3-kinase (PI3K)/AKT/mammalian target of rapamycin (mTOR) pathway: an emerging treatment strategy for squamous cell lung carcinoma. Cancer treatment reviews 40, 980–989 (2014). 25037117 10.1016/j.ctrv.2014.06.006

[b17] Yori J. L. *et al.* Combined SFK/mTOR inhibition prevents rapamycin-induced feedback activation of AKT and elicits efficient tumor regression. Cancer research 74, 4762–4771 (2014). 25023728 10.1158/0008-5472.CAN-13-3627 PMC4155007

[b18] Zardavas D., Fumagalli D. & Loi S. Phosphatidylinositol 3-kinase/AKT/mammalian target of rapamycin pathway inhibition: a breakthrough in the management of luminal (ER + /HER2-) breast cancers? Current opinion in oncology 24, 623–634 (2012). 22960556 10.1097/CCO.0b013e328358a2b5

[b19] Wu T. *et al.* Immunosuppressive drugs on inducing Ag-specific CD4(+)CD25(+)Foxp3(+) Treg cells during immune response *in vivo*. Transplant immunology 27, 30–38 (2012). 22613676 10.1016/j.trim.2012.05.001

[b20] Waickman A. T. & Powell J. D. mTOR, metabolism, and the regulation of T-cell differentiation and function. Immunological reviews 249, 43–58 (2012). 22889214 10.1111/j.1600-065X.2012.01152.x PMC3419491

[b21] Qu Y. *et al.* The effect of immunosuppressive drug rapamycin on regulatory CD4 + CD25 + Foxp3 + T cells in mice. Transplant immunology 17, 153–161 (2007). 17331841 10.1016/j.trim.2007.01.002

[b22] Saemann M. D., Haidinger M., Hecking M., Horl W. H. & Weichhart T. The multifunctional role of mTOR in innate immunity: implications for transplant immunity. American journal of transplantation: official journal of the American Society of Transplantation and the American Society of Transplant Surgeons 9, 2655–2661 (2009). 10.1111/j.1600-6143.2009.02832.x 19788500

[b23] Haidinger M. *et al.* A versatile role of mammalian target of rapamycin in human dendritic cell function and differentiation. J Immunol 185, 3919–3931 (2010). 20805416 10.4049/jimmunol.1000296

[b24] Weichhart T. *et al.* The TSC-mTOR signaling pathway regulates the innate inflammatory response. Immunity 29, 565–577 (2008). 18848473 10.1016/j.immuni.2008.08.012

[b25] Zhu L. *et al.* TSC1 controls macrophage polarization to prevent inflammatory disease. Nature communications 5, 4696 (2014). 10.1038/ncomms5696 25175012

[b26] Peranzoni E. *et al.* Myeloid-derived suppressor cell heterogeneity and subset definition. Curr Opin Immunol 22, 238–244 (2010). 20171075 10.1016/j.coi.2010.01.021

[b27] Chou Y. Y., Gao J. I., Chang S. F., Chang P. Y. & Lu S. C. Rapamycin inhibits lipopolysaccharide induction of granulocyte-colony stimulating factor and inducible nitric oxide synthase expression in macrophages by reducing the levels of octamer-binding factor-2. FEBS J 278, 85–96 (2011). 21114628 10.1111/j.1742-4658.2010.07929.x

[b28] Fox R. *et al.* PSGL-1 and mTOR regulate translation of ROCK-1 and physiological functions of macrophages. EMBO J 26, 505–515 (2007). 17245434 10.1038/sj.emboj.7601522 PMC1783463

[b29] Yang T. Y., Sun Y., Langnas A. N. & Zhao Y. Prolongation of allogeneic skin graft survival by injection of anti-Ly49A monoclonal antibody YE1/48. Clin Immunol 106, 148–154 (2003). 12672405 10.1016/s1521-6616(02)00041-4

[b30] Frias M. A. *et al.* mSin1 is necessary for Akt/PKB phosphorylation, and its isoforms define three distinct mTORC2s. Current biology: CB 16, 1865–1870 (2006). 16919458 10.1016/j.cub.2006.08.001

[b31] Jacinto E. *et al.* SIN1/MIP1 maintains rictor-mTOR complex integrity and regulates Akt phosphorylation and substrate specificity. Cell 127, 125–137 (2006). 16962653 10.1016/j.cell.2006.08.033

[b32] Sarbassov D. D. *et al.* Prolonged rapamycin treatment inhibits mTORC2 assembly and Akt/PKB. Mol Cell 22, 159–168 (2006). 16603397 10.1016/j.molcel.2006.03.029

[b33] Chen H. *et al.* Disruption of TSC1/2 signaling complex reveals a checkpoint governing thymic CD4 + CD25 + Foxp3 + regulatory T-cell development in mice. FASEB J 27, 3979–3990 (2013). 23882125 10.1096/fj.13-235408

[b34] Mazzoni A. *et al.* Myeloid suppressor lines inhibit T cell responses by an NO-dependent mechanism. J Immunol 168, 689–695 (2002). 11777962 10.4049/jimmunol.168.2.689

[b35] Yang L. *et al.* Abrogation of TGF beta signaling in mammary carcinomas recruits Gr-1 + CD11b + myeloid cells that promote metastasis. Cancer Cell 13, 23–35 (2008). 18167337 10.1016/j.ccr.2007.12.004 PMC2245859

[b36] Rees D. D., Palmer R. M., Hodson H. F. & Moncada S. A specific inhibitor of nitric oxide formation from L-arginine attenuates endothelium-dependent relaxation. Br J Pharmacol 96, 418–424 (1989). 2924084 10.1111/j.1476-5381.1989.tb11833.x PMC1854347

[b37] Pernicova I. & Korbonits M. Metformin–mode of action and clinical implications for diabetes and cancer. Nat Rev Endocrinol 10, 143–156 (2014). 24393785 10.1038/nrendo.2013.256

[b38] Inzucchi S. E., Lipska K. J., Mayo H., Bailey C. J. & McGuire D. K. Metformin in patients with type 2 diabetes and kidney disease: a systematic review. JAMA 312, 2668–2675 (2014). 25536258 10.1001/jama.2014.15298 PMC4427053

[b39] Saeedi R. *et al.* Metabolic actions of metformin in the heart can occur by AMPK-independent mechanisms. Am J Physiol Heart Circ Physiol 294, H2497–2506 (2008). 18375721 10.1152/ajpheart.00873.2007

[b40] Cheong J. H. *et al.* Dual inhibition of tumor energy pathway by 2-deoxyglucose and metformin is effective against a broad spectrum of preclinical cancer models. Mol Cancer Ther 10, 2350–2362 (2011). 21992792 10.1158/1535-7163.MCT-11-0497 PMC3237863

[b41] Pierotti M. A. *et al.* Targeting metabolism for cancer treatment and prevention: metformin, an old drug with multi-faceted effects. Oncogene 32, 1475–1487 (2013). 22665053 10.1038/onc.2012.181

[b42] Janzer A. *et al.* Metformin and phenformin deplete tricarboxylic acid cycle and glycolytic intermediates during cell transformation and NTPs in cancer stem cells. Proc Natl Acad Sci USA 111, 10574–10579 (2014). 25002509 10.1073/pnas.1409844111 PMC4115496

[b43] Kirsch A. H. *et al.* The mTOR-inhibitor rapamycin mediates proteinuria in nephrotoxic serum nephritis by activating the innate immune response. American journal of physiology. Renal physiology 303, F569–575 (2012). 22696604 10.1152/ajprenal.00180.2012

[b44] Weichhart T. *et al.* Inhibition of mTOR blocks the anti-inflammatory effects of glucocorticoids in myeloid immune cells. Blood 117, 4273–4283 (2011). 21368289 10.1182/blood-2010-09-310888

[b45] Brouard S. *et al.* Comparative transcriptional and phenotypic peripheral blood analysis of kidney recipients under cyclosporin A or sirolimus monotherapy. American journal of transplantation: official journal of the American Society of Transplantation and the American Society of Transplant Surgeons 10, 2604–2614 (2010). 10.1111/j.1600-6143.2010.03302.x 21114641

[b46] Dugast A. S. *et al.* Myeloid-derived suppressor cells accumulate in kidney allograft tolerance and specifically suppress effector T cell expansion. J Immunol 180, 7898–7906 (2008). 18523253 10.4049/jimmunol.180.12.7898

[b47] Zhang W., Liang S., Wu J. & Horuzsko A. Human inhibitory receptor immunoglobulin-like transcript 2 amplifies CD11b + Gr1 + myeloid-derived suppressor cells that promote long-term survival of allografts. Transplantation 86, 1125–1134 (2008). 18946352 10.1097/TP.0b013e318186fccd PMC2668611

[b48] Inoki K., Kim J. & Guan K. L. AMPK and mTOR in cellular energy homeostasis and drug targets. Annual review of pharmacology and toxicology 52, 381–400 (2012). 10.1146/annurev-pharmtox-010611-134537 22017684

[b49] Laplante M. & Sabatini D. M. mTOR signaling in growth control and disease. Cell 149, 274–293 (2012). 22500797 10.1016/j.cell.2012.03.017 PMC3331679

[b50] Warburg O., Wind F. & Negelein E. The Metabolism of Tumors in the Body. The Journal of general physiology 8, 519–530 (1927). 19872213 10.1085/jgp.8.6.519 PMC2140820

[b51] Calvano S. E. *et al.* A network-based analysis of systemic inflammation in humans. Nature 437, 1032–1037 (2005). 16136080 10.1038/nature03985

[b52] Everts B. *et al.* Commitment to glycolysis sustains survival of NO-producing inflammatory dendritic cells. Blood 120, 1422–1431 (2012). 22786879 10.1182/blood-2012-03-419747 PMC3423780

[b53] Wang Y. *et al.* Tuberous sclerosis 1 (Tsc1)-dependent metabolic checkpoint controls development of dendritic cells. Proc Natl Acad Sci USA 110, E4894–4903 (2013). 24282297 10.1073/pnas.1308905110 PMC3864282

[b54] Goodarzi M. O. & Bryer-Ash M. Metformin revisited: re-evaluation of its properties and role in the pharmacopoeia of modern antidiabetic agents. Diabetes, obesity & metabolism 7, 654–665 (2005). 10.1111/j.1463-1326.2004.00448.x 16219009

[b55] Zhou G. *et al.* Role of AMP-activated protein kinase in mechanism of metformin action. The Journal of clinical investigation 108, 1167–1174 (2001). 11602624 10.1172/JCI13505 PMC209533

[b56] Tanaka T., Sue F. & Morimura H. Feed-forward activation and feed-back inhibition of pyruvate kinase type L of rat liver. Biochemical and biophysical research communications 29, 444–449 (1967). 6076246 10.1016/0006-291x(67)90477-9

[b57] Vasamsetti S. B. *et al.* Metformin inhibits monocyte-to-macrophage differentiation via AMPK-mediated inhibition of STAT3 activation: potential role in atherosclerosis. Diabetes 64, 2028–2041 (2015). 25552600 10.2337/db14-1225

[b58] Marigo I. *et al.* Tumor-induced tolerance and immune suppression depend on the C/EBPbeta transcription factor. Immunity 32, 790–802 (2010). 20605485 10.1016/j.immuni.2010.05.010

[b59] Qu Y. *et al.* 2-Gy whole-body irradiation significantly alters the balance of CD4 + CD25- T effector cells and CD4 + CD25 + Foxp3 + T regulatory cells in mice. Cellular & molecular immunology 7, 419–427 (2010). 20871628 10.1038/cmi.2010.45 PMC4002961

[b60] Angulo I. *et al.* Nitric oxide-producing CD11b(+)Ly-6G(Gr-1)(+)CD31(ER-MP12)(+) cells in the spleen of cyclophosphamide-treated mice: implications for T-cell responses in immunosuppressed mice. Blood 95, 212–220 (2000). 10607705

[b61] Hou Y. *et al.* The inhibitory effect of IFN-gamma on protease HTRA1 expression in rheumatoid arthritis. J Immunol 193, 130–138 (2014). 24907345 10.4049/jimmunol.1302700

