# Supplementary material for: mTOR masters monocytic myeloid-derived suppressor cells in mice with allografts or tumors
Source: Sci Rep. 2016 Feb 1;6:20250. doi: 10.1038/srep20250 (PMC4735296; doi:10.1038/srep20250)
Supplement: Supplementary Information [file srep20250-s1.pdf]

mTOR masters monocytic myeloid-derived suppressor cells in mice with allografts or tumors

Tingting Wu[1,*], Yang Zhao[1,*], Hao Wang[2,*], yang Li[1], Lijuan Shao[1,2], Ruoyu Wang[2], Jun Lu[3], Zhongzhou, Yang[4,#], Junjie Wang[2,#], and Yong Zhao[1,#]

Suppl. Fig. 1

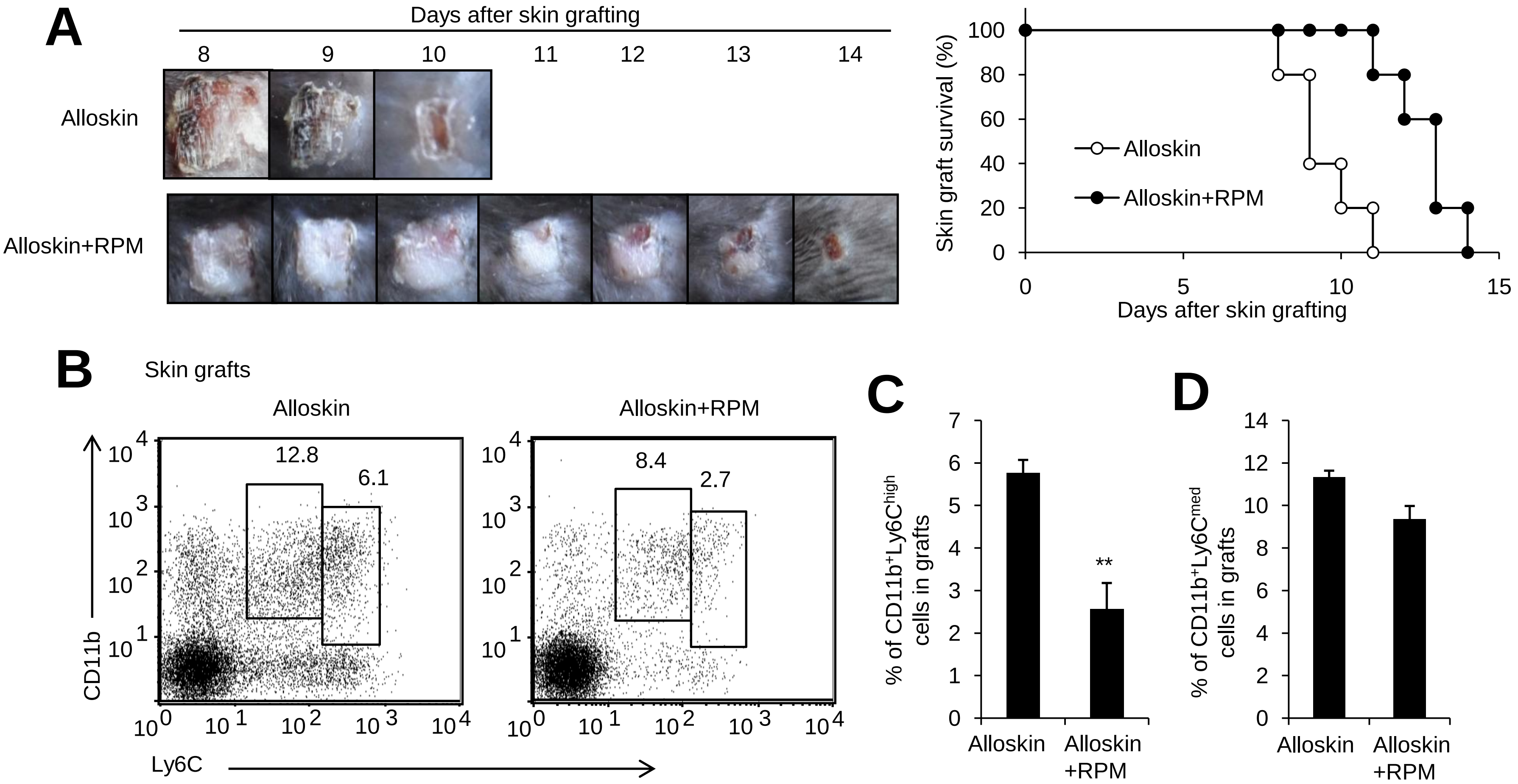

Supplementary figure 1. RPM treatment significantly delayed alloskin graft rejection and decreased CD11b$^+$Ly6C^{hi} M-MDSCs in alloskin grafts.

C57BL/6 mice were transplanted with alloskin grafts and intraperitoneally administered with RPM at a dose of 1.5mg/kg from day -3 to day 7. **(A)**. Macroscopic pictures of alloskin grafts were collected at different time points and graft survival rates were compared by the log-rank test. **(B)**. Typical example of flow cytometry analysis. Cells were isolated from alloskin grafts at day 7 as described in materials and methods and analyzed by flow cytometry. M-MDSCs were gated as CD11b$^+$Ly6C^{hi} cell subset and G-MDSCs were gated as CD11b$^+$Ly6C^{med} cell subset. The percentages of M-MDSCs (**C**) and G-MDSCs (**D**) in alloskin grafts. Each group included from 4 to 6 mice and at least three independent experiments showed similar results. **$p<0.01$compared between the indicated groups.

Suppl. Fig. 2

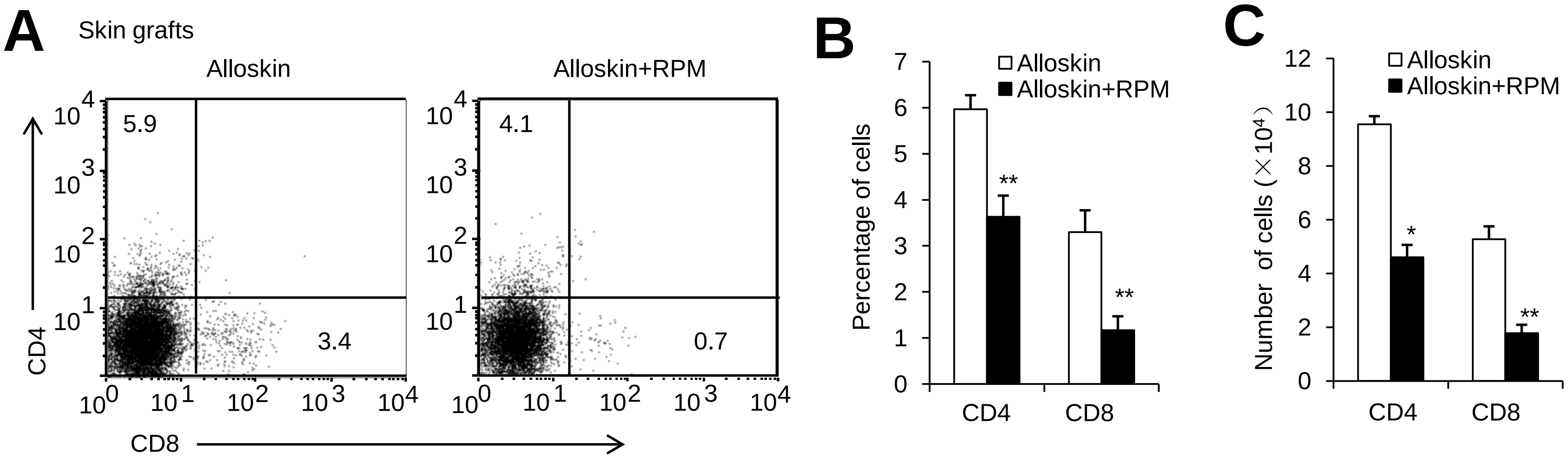

Supplementary figure 2. RPM treatment decreased CD4 and CD8 T cells in alloskin grafts.
C57BL/6 mice were transplanted with alloskin grafts and intraperitoneally administered with RPM at a dose of 1.5mg/kg from day -3 to day 7. **(A)**. Typical example of flow cytometry analysis. Cells were isolated from alloskin grafts at day 7 as described in materials and methods and analyzed by flow cytometry. The percentages **(B)** and cell numbers **(C)** of CD4 and CD8 T cells in alloskin grafts. Each group included from 4 to 6 mice and at least three independent experiments showed similar results. *$p<0.05$ and **$p<0.01$compared between the indicated groups.

Suppl. Fig. 3

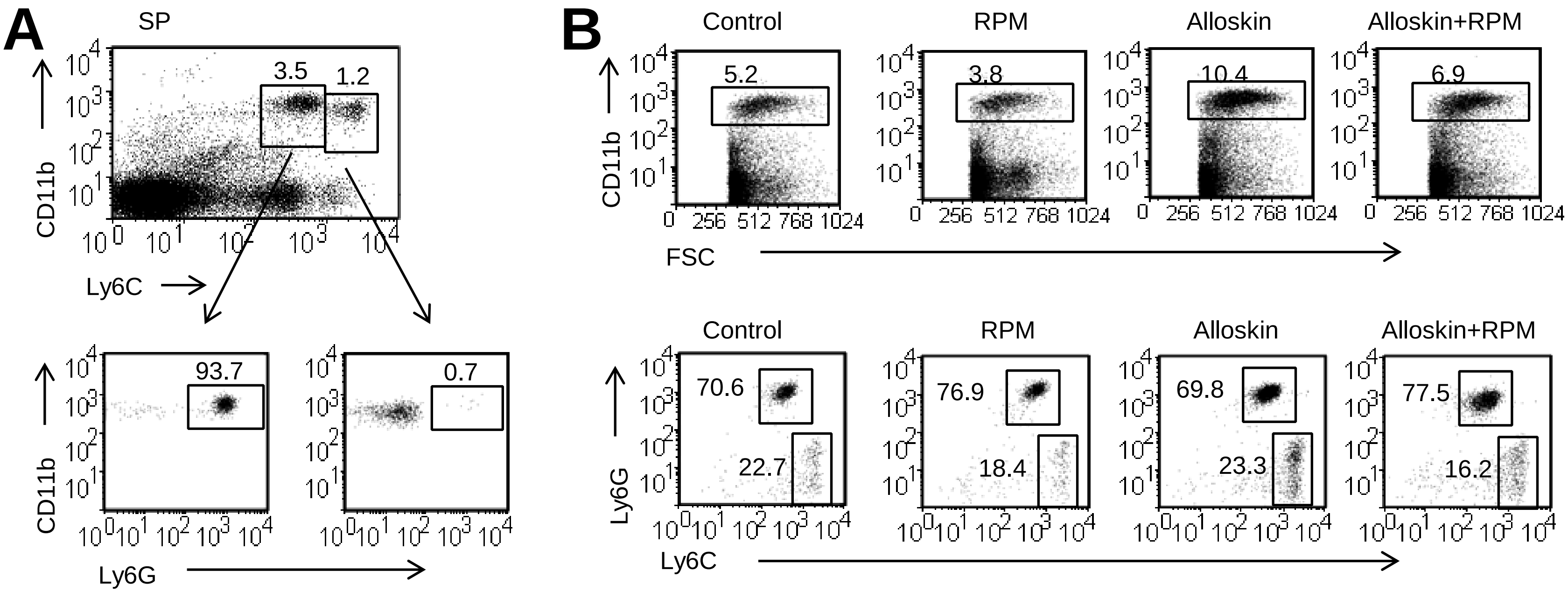

Supplementary figure 3. M-MDSCs and G-MDSCs can be distinguished directly by their different expression levels of Ly6C.

(A). Analysis of Ly6G expression in CD11b^{+}Ly6C^{med} cell subset (lower, left) and CD11b^{+}Ly6C^{hi} cell subset (lower, right) in C57BL/6 mice demonstrated that most of CD11b^{+}Ly6C^{med} cells expressed Ly6G, whereas CD11b^{+}Ly6C^{hi} cells are Ly6G negative cells. **(B)**. Analysis of Ly6C^{med}Ly6G^{hi} G-MDSCs and Ly6C^{hi}Ly6G^{neg} M-MDSCs when gated in CD11b^{+} cells demonstrated that RPM treatment significantly decreased Ly6C^{hi}Ly6G^{neg} M-MDSCs.

Suppl. Fig. 4

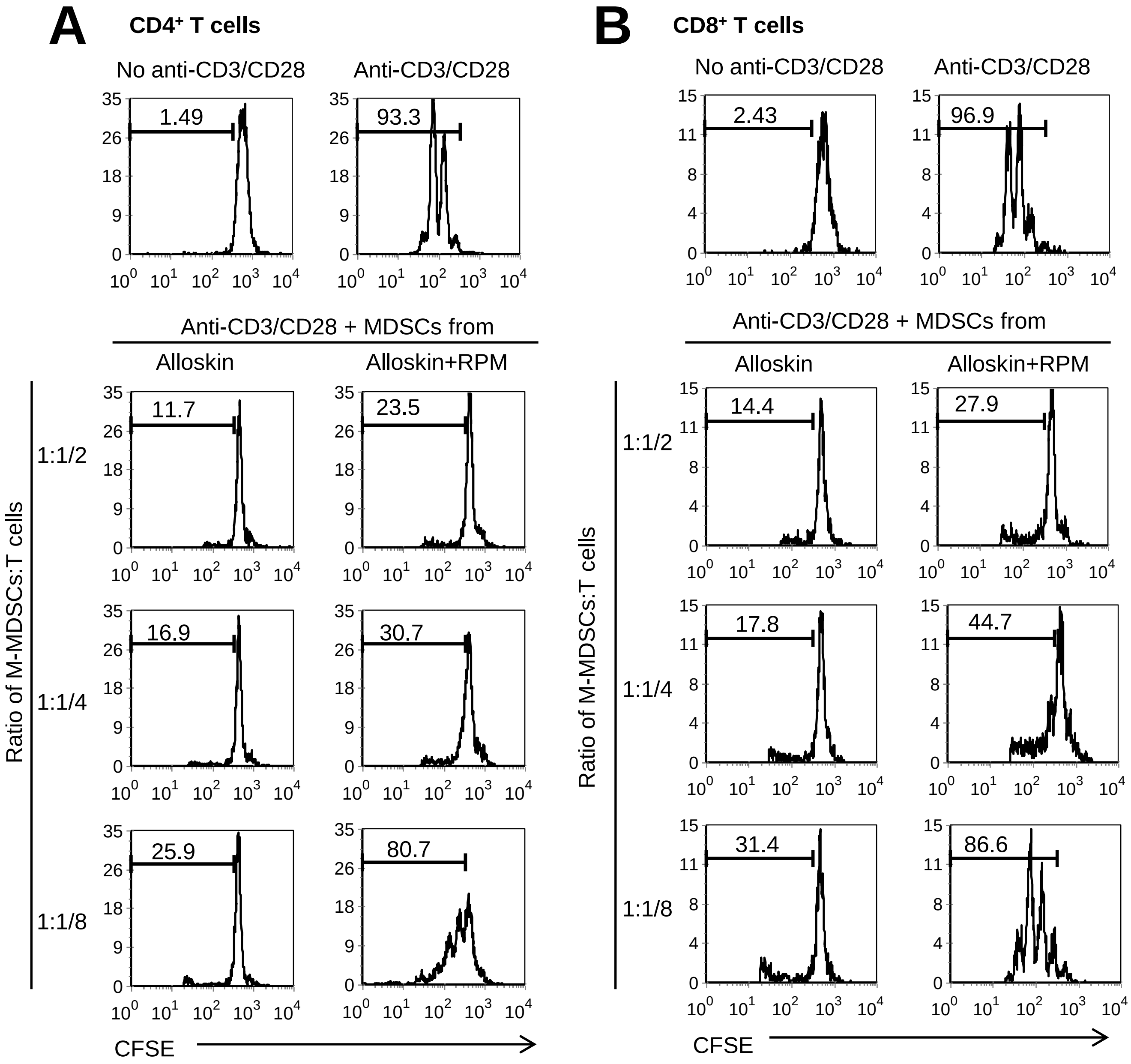

Supplementary figure 4. RPM treatment significantly inhibited function of CD11b$^+$Ly6G$^-$ M-MDSCs. CFSE-labeled T cells were stimulated in vitro with anti-CD3/CD28 mAbs and CD11b$^+$ Ly6C^{hi} M-MDSCs from spleens of skin-grafted recipients were added at different ratios. After culture for 3 days, cells were stained with CD4 and CD8, and CFSE signal of gated lymphocytes was analyzed. CFSE intensity of CD4$^+$ and CD8$^+$ T cells is proportional to the number of cell divisions. Stimulated T cells showed multiple CFSE dilution peaks, which were abolished when M-MDSCs were added, and the inhibition of CFSE dilution was dose-dependent. One experiment representative of three independent experiments is shown.

Suppl. Fig. 5

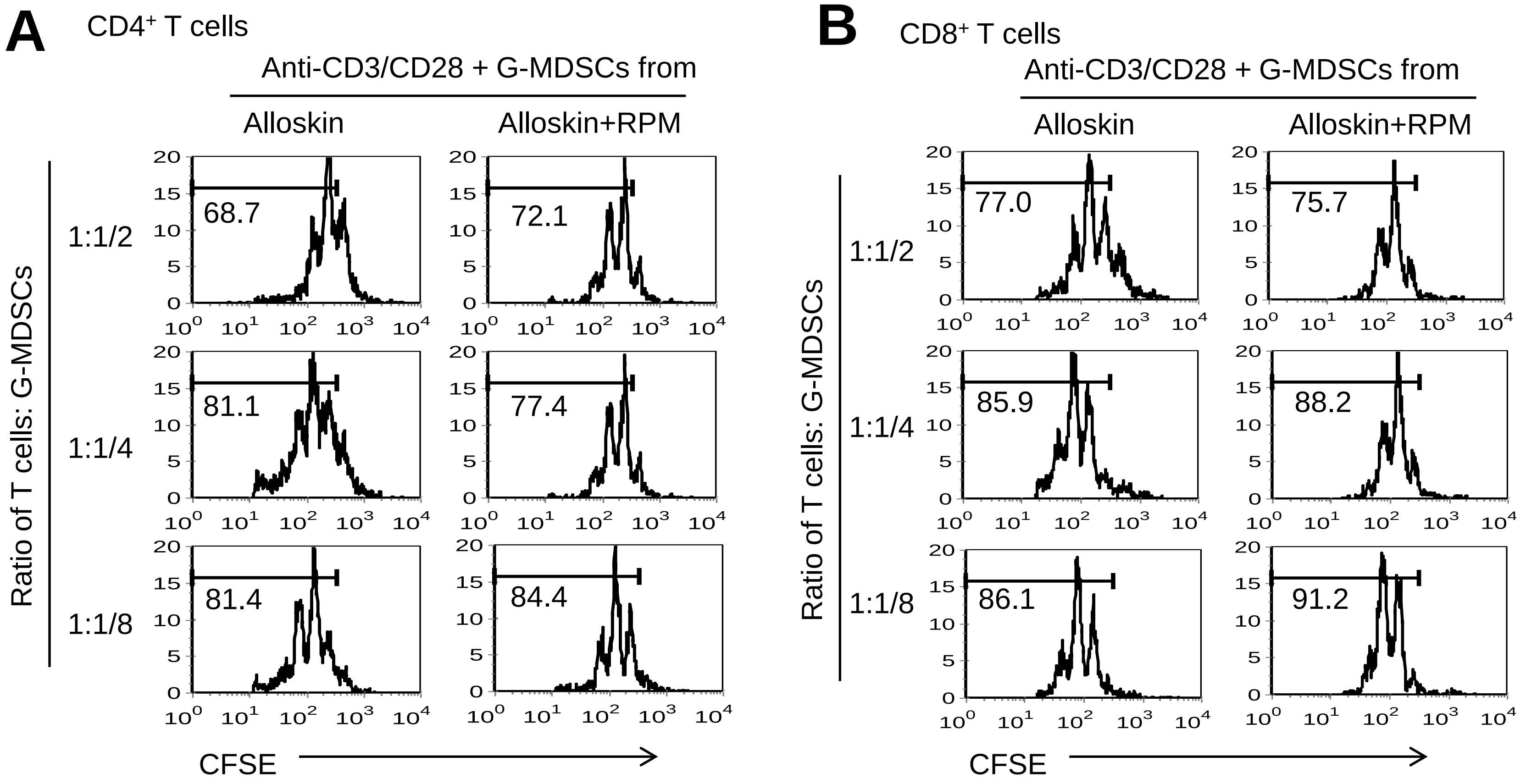

Supplementary figure 5. G-MDSCs isolated from alloskin-grafted mice failed to show strong suppression on the proliferation of T cells

CFSE-labeled T cells were stimulated in vitro by anti-CD3/CD28 mAbs and $CD11b^{+}$ $Ly6C^{med}$ G-MDSCs from spleens of recipients were added at different ratios. After culture for 3 days, cells were stained with anti-CD4 and anti-CD8 mAbs, and CFSE signal of gated CD4+ T cells (**A**) and CD8+ T cells (**B**) was analyzed. G-MDSCs isolated from alloskin-grafted mice did not significantly inhibit the proliferation of T cells. One experiment representative of three independent experiments is shown.

Suppl. Fig. 6

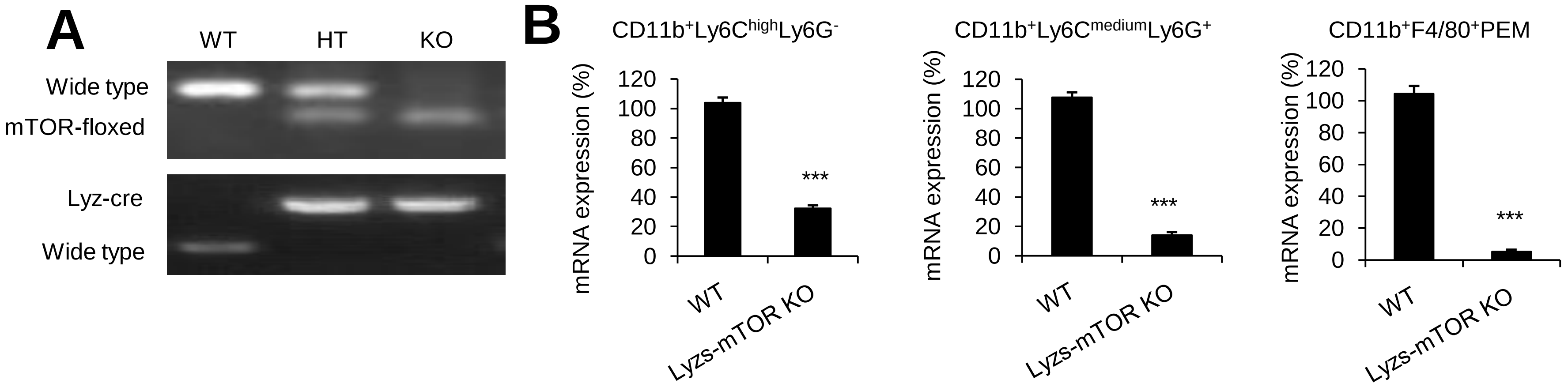

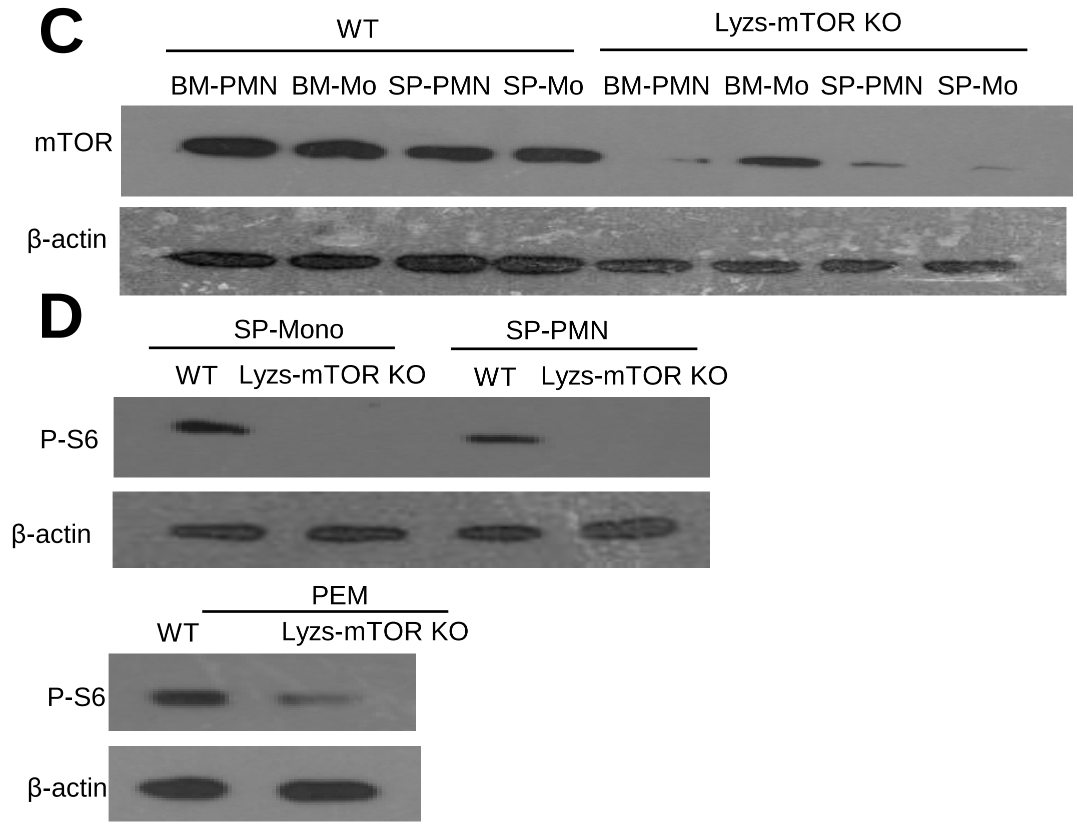

Supplementary figure 6. Genotyping of WT, HT and Lyzs-mTOR KO mice.

(A). Genotype of WT is lyzs-cre^{-} mTOR$^{+/+}$, Genotype of Heterozygous (HT) is lyzs-cre^{+}mTOR$^{loxp/+}$ and Genotype of lyzs-mTOR KO is Lyzs-cre^{+}mTOR$^{loxp/loxp}$. **(B).** The mRNA expressions of mTOR in splenic CD11b^{+}Ly6C^{high}Ly6G cells,CD11b^{+}Ly6C^{medium}Ly6G^{+} cells and CD11b^{+}F4/80^{+} PEMs were detected by qPCR. **(C)**. The protein expressions of mTOR in splenic and bone marrow (BM) monocytes/macrophages (CD11b^{+}Ly6C^{high}Ly6G^{-} cells) and PMNs (CD11b^{+}Ly6C^{medium}Ly6G^{+} cells) were detected by western blots. **(D)**. The down stream signaling of mTOR in splenic monocytes/macrophages (CD11b^{+}Ly6C^{high}Ly6G^{-} cells) and PMNs (CD11b^{+}Ly6C^{medium}Ly6G^{+} cells) were detected by western blots. p-S6 expression is significantly decreased in splenic myeloid cells of Lyzs-mTOR KO mice.

Suppl. Fig. 7

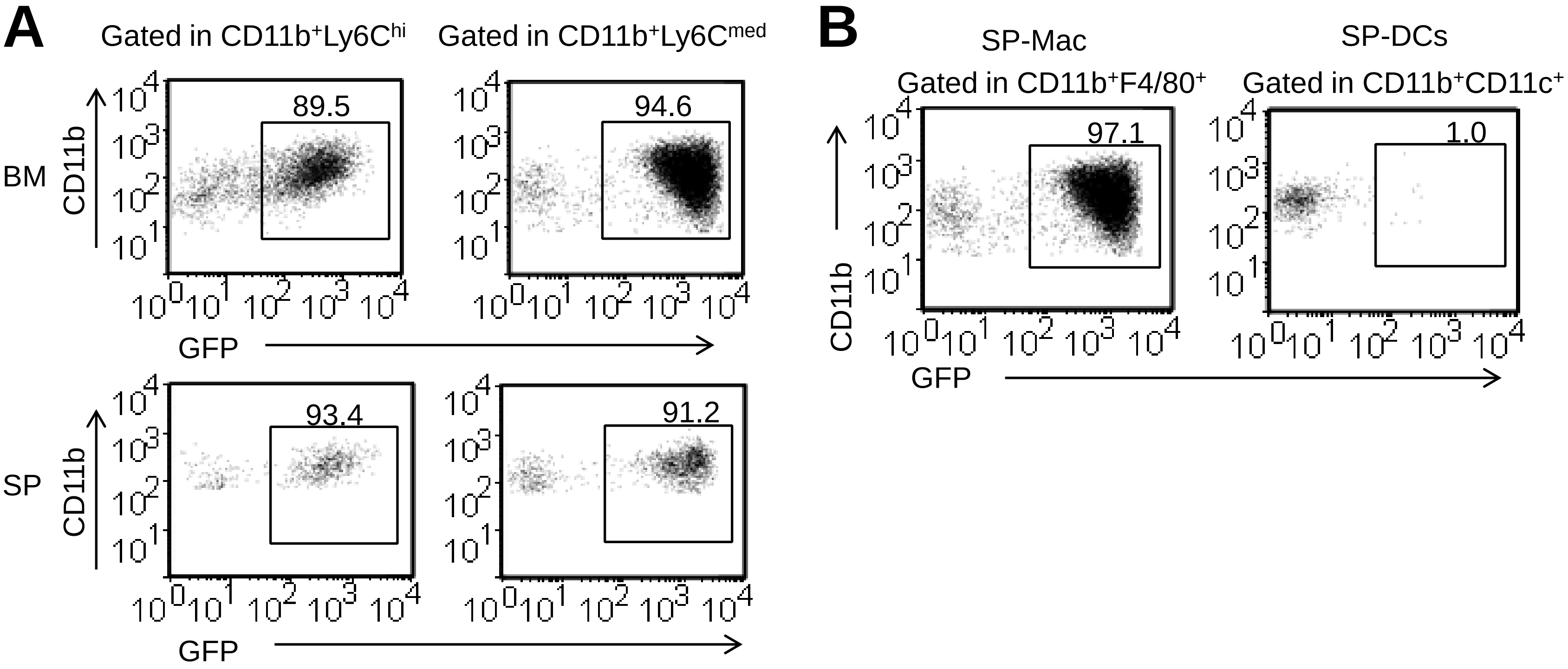

Supplementary figure 7. Lyzs expression in different cell subsets of myeloid cells in Lyzs-GFP reporter mice.

(A). The lyzs-GFP expression in CD11b$^+$Ly6C^{high} cells and CD11b$^+$Ly6C^{medium} cells in the bone marrow and spleens of lyzs-GFP mice. **(B)**. The lyzs-GFP expression in CD11b$^+$F4/80$^+$ macropahges and CD11b$^+$CD11c$^+$ dendritic cells in the spleen of lyzs-GFP reporter mice.

Suppl. Fig. 8

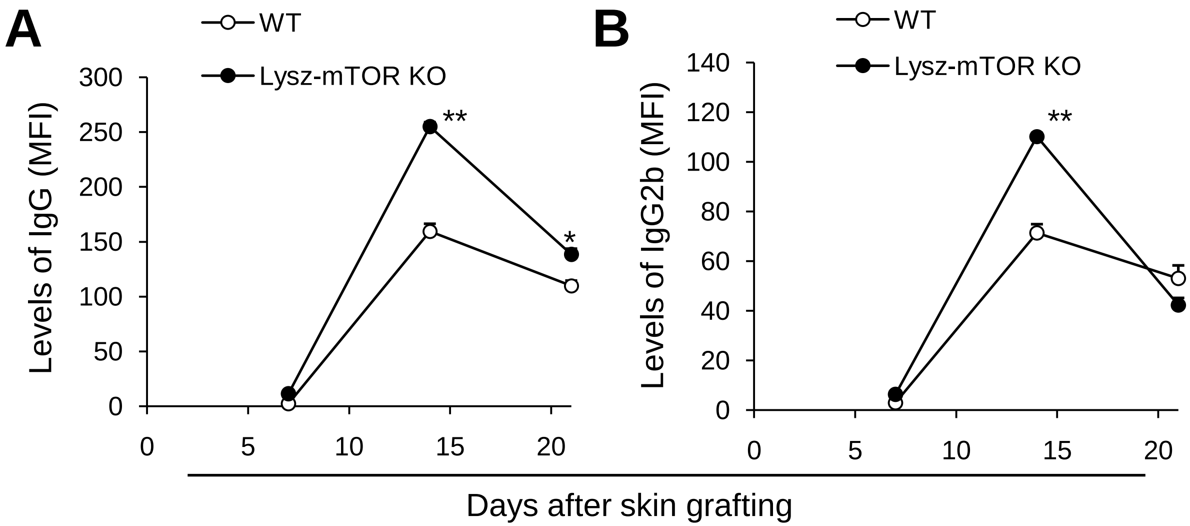

Supplementary figure 8. The levels of anti-donor IgG and IgG2b in mTOR KO recipients were higher than WT recipients after 2 weeks post transplantation. The IgG isotype of anti-donor alloreactive Abs in sera of alloskin-grafted WT and lyzs-mTOR KO mice were measured by flow cytometry as described in Materials and Methods. **(A)**. The kinetics of IgG production in the sera after alloskin transplantation. **(B)**. The kinetics of IgG2b production in sera after alloskin transplantation.

Suppl. Fig. 9

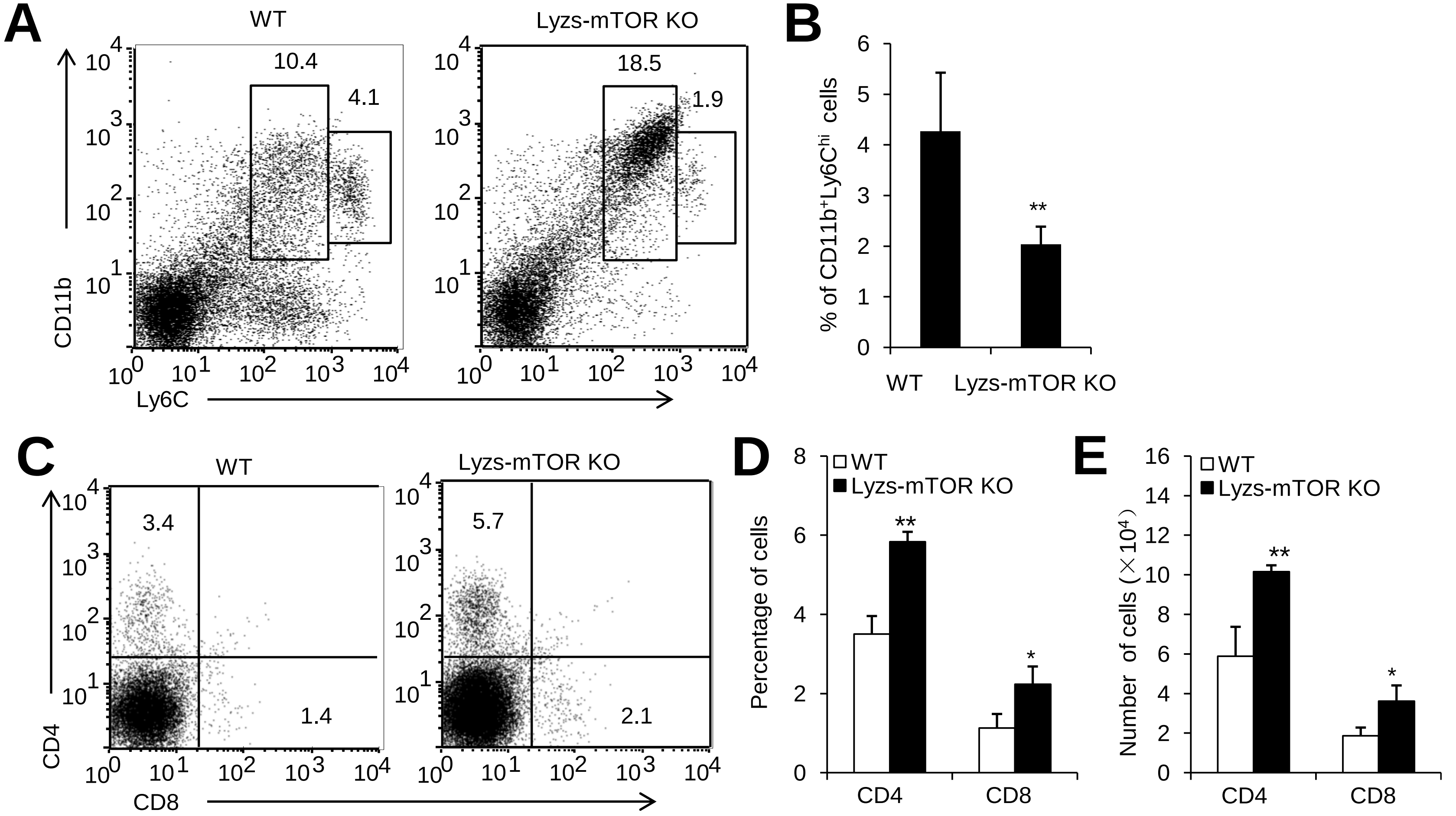

Supplementary figure 9. The infiltrated CD11b$^+$Ly6C^{hi} M-MDSCs, CD4$^+$ and CD8$^+$ T cells in alloskin grafts in WT and Lyzs-mTOR KO mice

WT and Lyzs-mTOR KO mice were transplanted with alloskin grafts and grafts were analyzed at day 7. Cells were isolated from alloskin grafts as described in materials and methods and analyzed by flow cytometry. M-MDSCs were gated as CD11b$^+$Ly6C^{hi} cell subset and G-MDSCs were gated as CD11b$^+$Ly6C^{med} cell subset. **(A).** Typical example of flow cytometry analysis. **(B).**The percentages of M-MDSCs in alloskin grafts. **(C).** Typical example of flow cytometry analysis for CD4$^+$ and CD8$^+$ T cells in grafts. **(D).**The mean percentages of CD4$^+$ and CD8$^+$ T cells in alloskin grafts. **(E).**The total cell numbers of CD4$^+$ and CD8$^+$ T cells in alloskin grafts. Each group included from 4 to 6 mice and at least three independent experiments showed similar results. *p<0.05, **p<0.01 compared between the indicated groups.

Suppl. Fig . 10

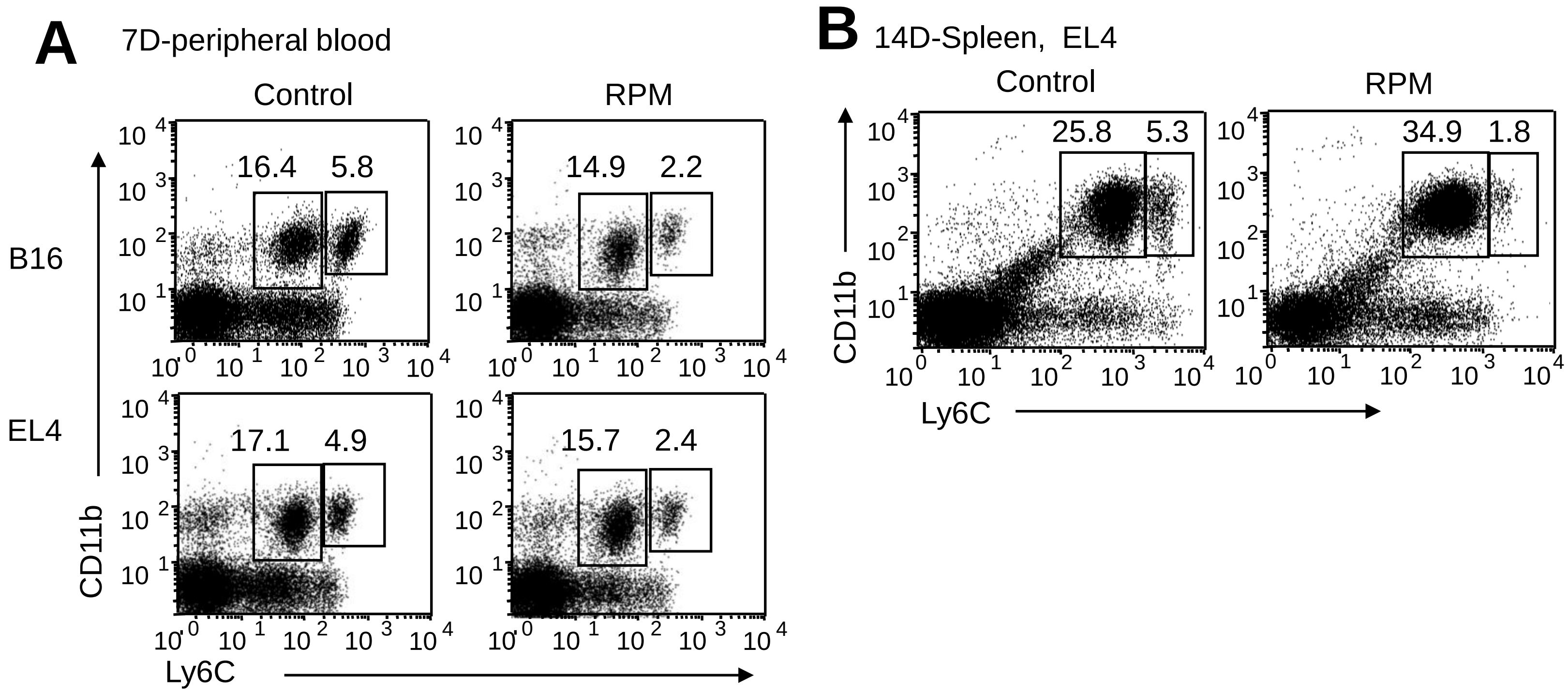

Supplementary figure 10. RPM treatment significantly decreased CD11b$^+$Ly6C^{high} M-MDSCs in the peripheral blood and spleens of B16- or EL4-bearing B6 mice.

C57BL/6 recipients with the same age and sex were injected s.c. with 1.5 $\times 10^6$ EL4 lymphoma or 5 $\times 10^5$ B16 melanoma. And then, these mice were intraperitoneally administered with RPM at a dose of 1.5 mg/kg from day 0 to day 14. Typical example of flow cytometry analysis of MDSCs from peripheral blood of tumor-loaded mice at day 7 (**A**) or from spleen of EL4 tumor-loaded mice at day 14 (**B**). Cells were stained with anti-CD11b and anti-Ly6C antibodies. M-MDSCs were gated as CD11b$^+$Ly6C^{high} cell subset and G-MDSCs were gated as CD11b$^+$Ly6C^{med} cell subset. One experiment representative of three independent experiments is shown.

Suppl. Fig . 11

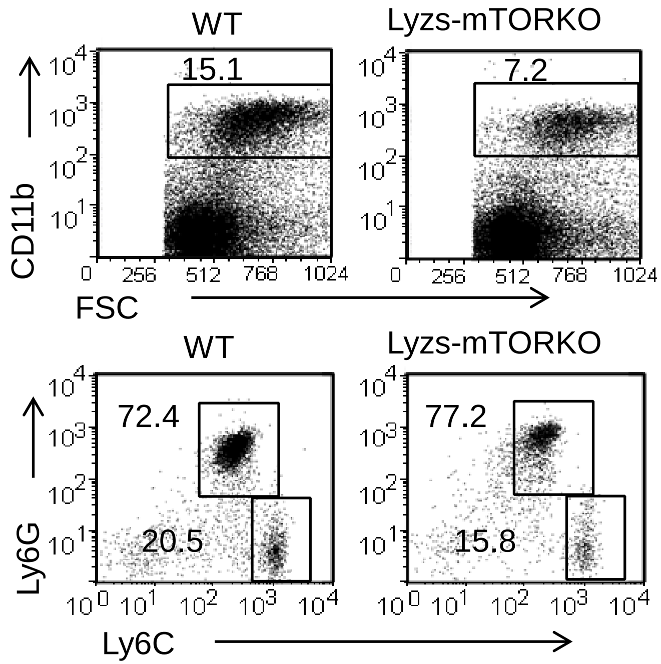

Supplementary figure 11. The levels of M-MDSCs and G-MDSCs in tumor-bearing WT and Lyzs-mTOR KO mice.

Analysis of $CD11b^{+}Ly6C^{hi}Ly6G^{-}$ M-MDSCs and $CD11b^{+}Ly6C^{med}Ly6G^{+}$ G-MDSCs from spleens of tumor-loaded WT or Lyzs-mTOR KO mice at day 10 after tumor implantation.

Suppl. Fig. 12

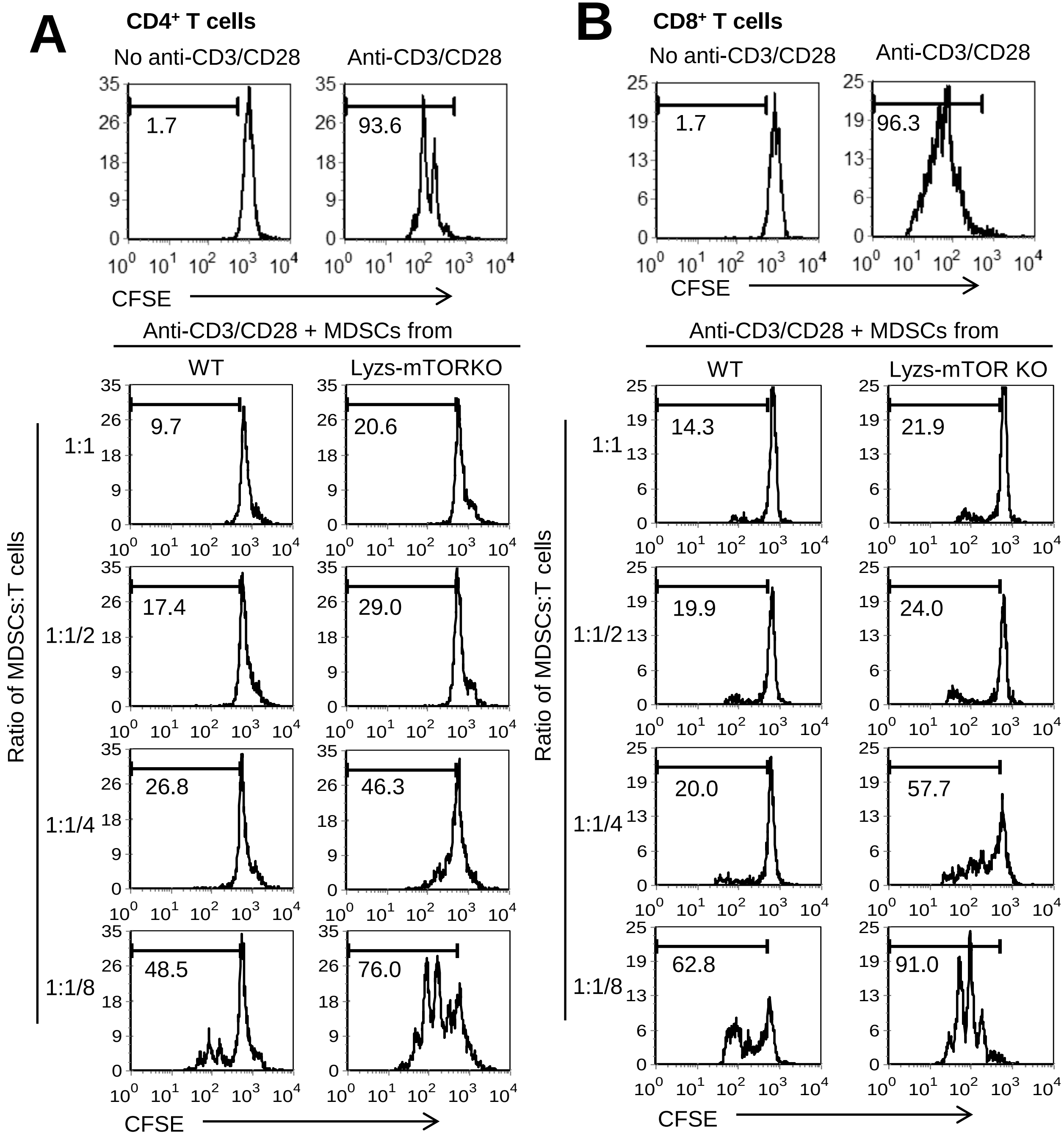

Supplementary figure 12. The inhibitory effects on T cell proliferation of WT and mTOR KO M-MDSCs isolated from tumor-bearing WT and Lyzs-mTOR KO mice.

The sorted $CD11b^{+}Ly6C^{hi}Ly6G^{-}$ M-MDSCs from spleens of WT and Lyzs-mTOR KO mice which loaded EL-4 tumor for 10 days were added at different ratios in T cell proliferation system for 72h. Typical examples of flow cytometry analysis of CFSE-labeled T cell proliferation were shown.

Suppl. Fig . 13

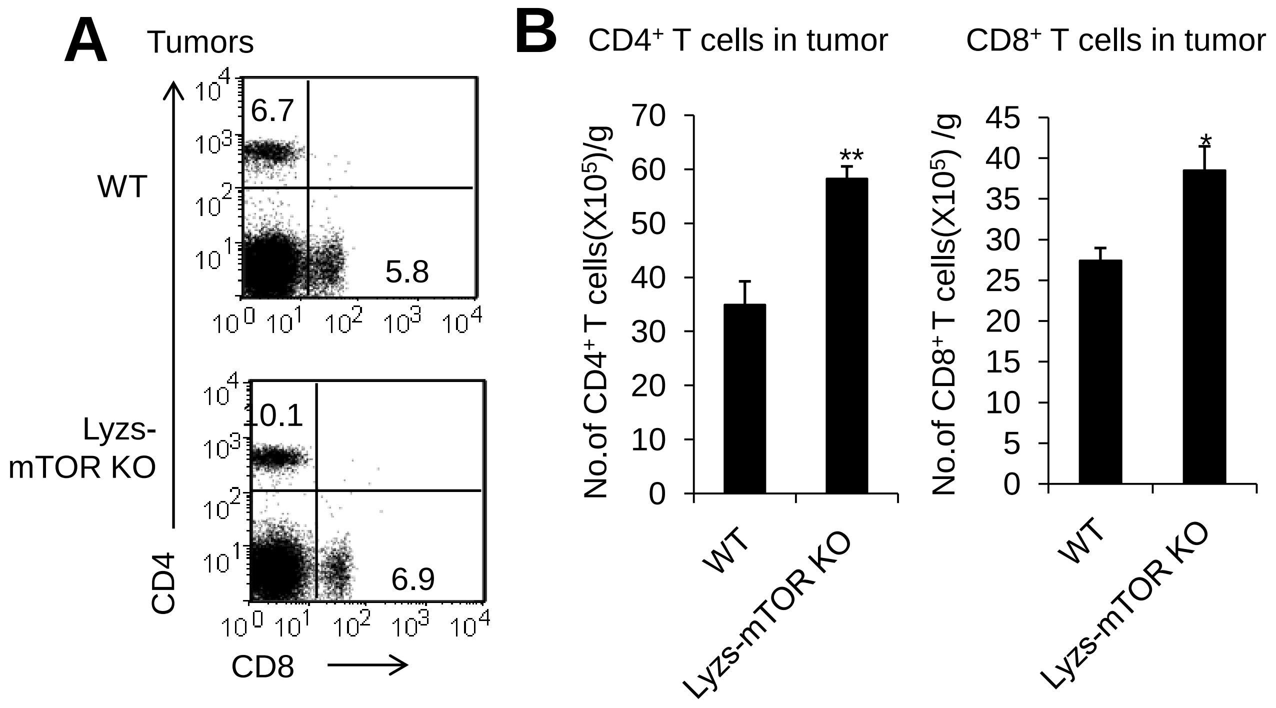

Supplementary figure 13. The infiltrated CD4$^+$T cells and CD8$^+$T cells in tumors of WT and Lyzs-mTOR KO mice.

(A). Flow cytometry analysis of CD4 and CD8 T cells in tumor tissues of WT or Lyzs-mTOR KO mice at day 10. The numbers in FACS plots represent the percentages of cells in the gates. **(B)**. The cell numbers of CD4$^+$ and CD8$^+$ T cells in tumor tissues from EL-4-loaded WT or Lyzs-mTOR KO mice at day 10. Each group included from 4 to 6 mice and at least three independent experiments showed similar results. *p<0.05 and **p<0.01compared between the indicated groups.

Suppl. Fig. 14

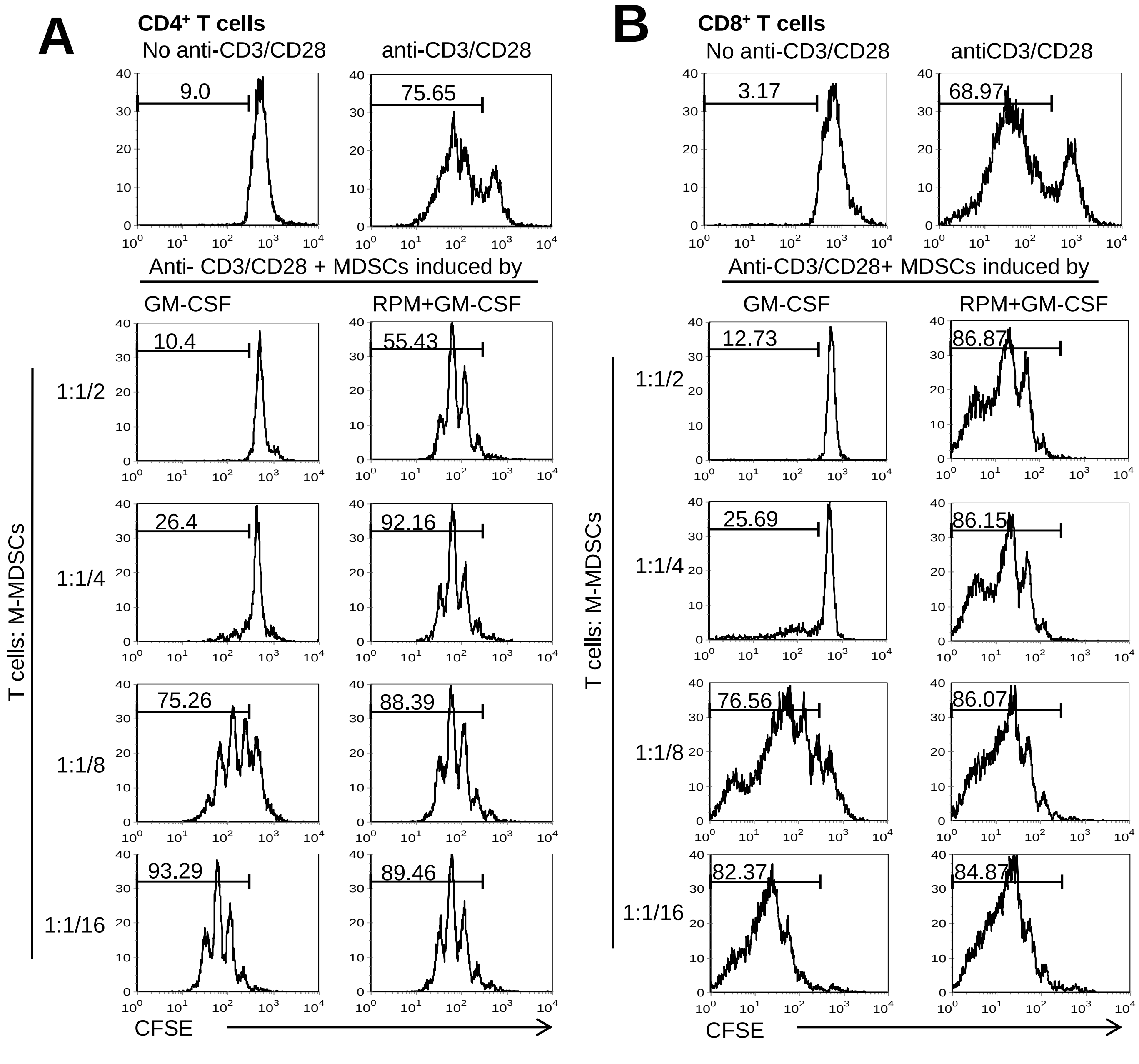

Supplementary figure 14. The inhibitory effects on T cell proliferation of GM-CSF-induced M-MDSCs in the presence or absence of RPM.

M-MDSCs induced from C57BL/6 bone marrow cells by GM-CSF in the presence or absence of RPM. Sorted CD11b^{+}Ly6C^{hi}Ly6G^{-} M-MDSCs were added at different ratios in T cell proliferation system for 72h. Typical examples of flow cytometry analysis of CFSE-labeled T cell proliferation were shown.

Suppl. Fig. 15

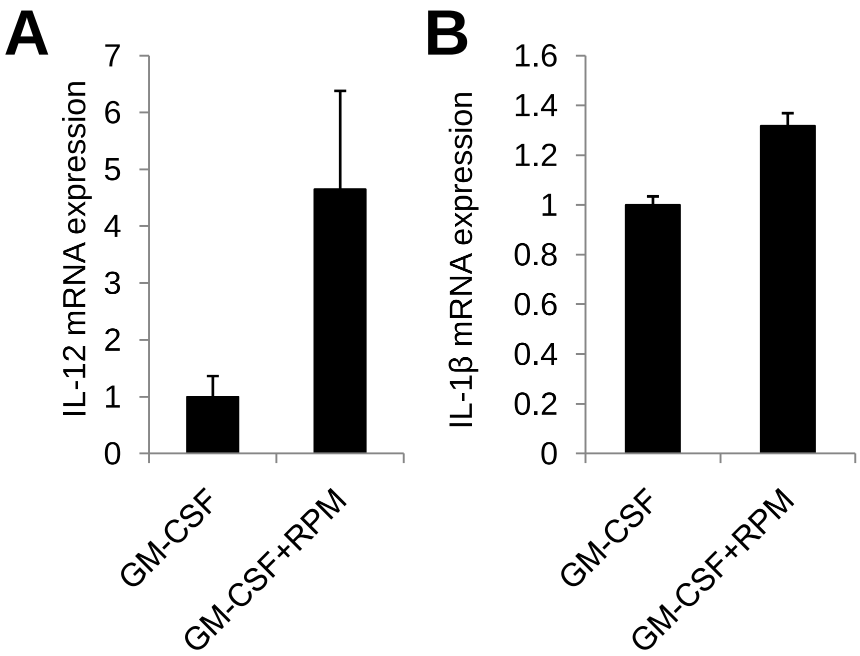

Supplementary figure 15. The inflammatory cytokine expression of GM-CSF-induced M-MDSCs in the presence or absence of RPM.

M-MDSCs induced from C57BL/6 bone marrow cells by GM-CSF in the presence or absence of RPM. The expression of inflammatory cytokines in the sorted CD11b^{+}Ly6C^{hi}Ly6G^{-} M-MDSCs were detected by real-time PCR.

Suppl. Fig. 16

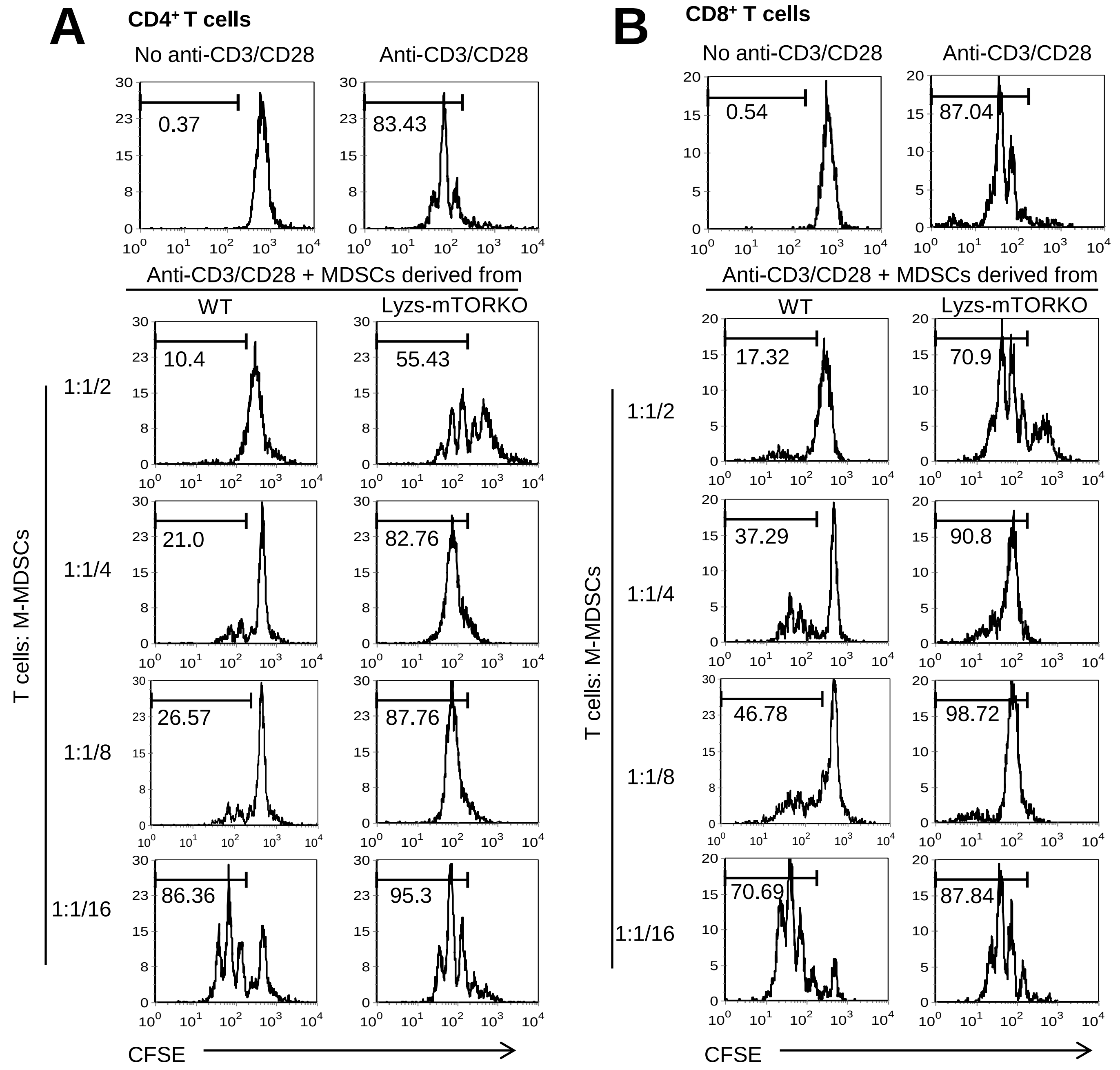

Supplementary figure 16. The inhibitory effects on T cell proliferation of GM-CSF-induced WT and mTOR KO M-MDSCs. M-MDSCs induced from WT and Lyzs-mTOR KO bone marrow cells by 40 ng/ml GM-CSF for 4 days. Sorted $CD11b^+Ly6C^{hi}Ly6G^-$ M-MDSCs were added at different ratios in T cell proliferation system for 72h. Typical examples of flow cytometry analysis of CFSE-labeled T cell proliferation were shown.

Suppl. Fig. 17

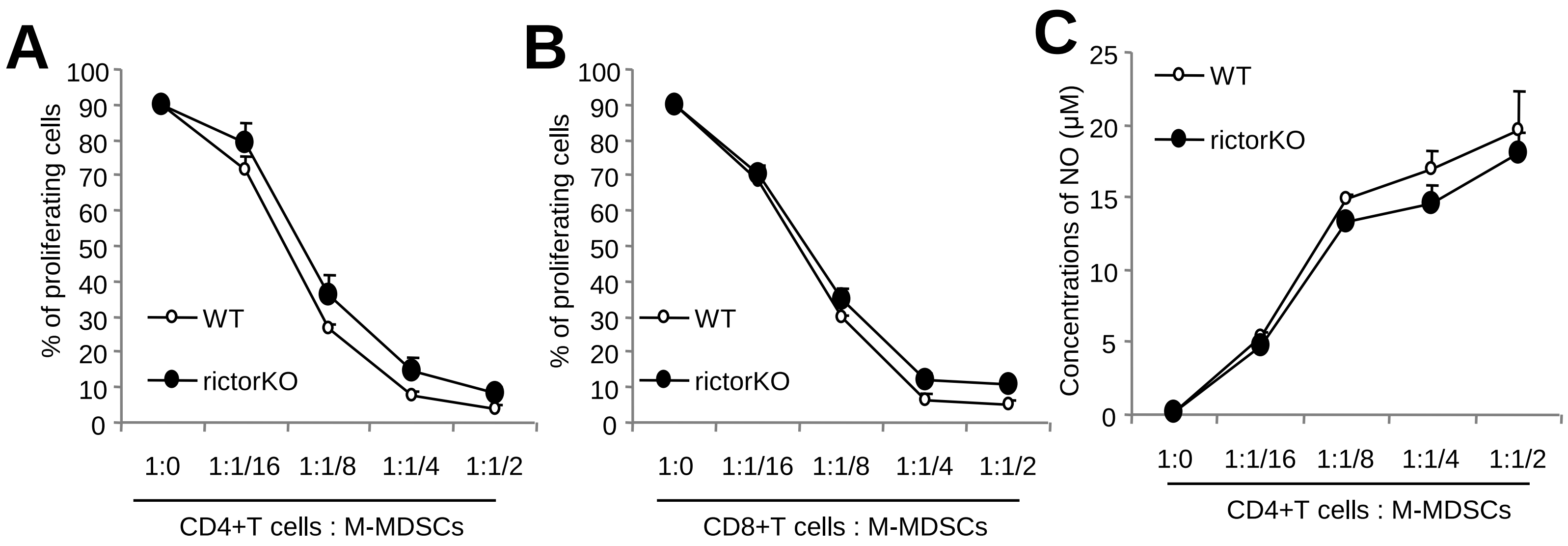

Supplementary figure 17. mTORC2 is not necessary for M-MDSC immunosuppressive function and NO production.

Sorted $CD11b^{+}Ly6C^{hi}$ $Ly6G^{-}$ M-MDSCs induced from WT or Lyzs-rictor KO bone marrow cells after 4 days of culture in the presence of 40 ng/ml GM-CSF were added to T cell proliferation system at different ratio for 72h. CFSE signal of gated $CD4^{+}$ T cells (**A**) and $CD8^{+}$ T cells (**B**) was analyzed. The extent of cell proliferation was quantified by ModFit LT software V3.0 (Verity Software House, Inc.). **(C)**. Nitrite concentrations in the cell culture medium of immunosuppressive assays at day 3 was measured as described in materials and methods.

Suppl. Fig. 18

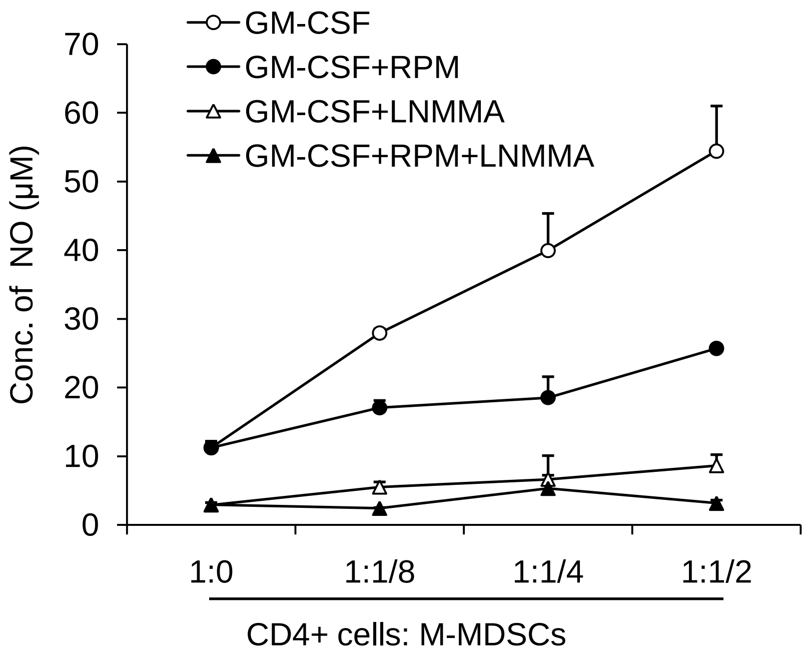

Supplementary figure 18. L-NMMA almost completely blocked NO production by M-MDSCs in the co-culture system.

GM-CSF-induced M-MDSCs were co-cultured with T cells in the presence of anti-CD3/28 mAbs, 2 mM L-NMMA was added in the T cell suppression assay and nitrite concentrations of cell culture medium at day 3 was measured as described in materials and methods.

Suppl. Fig. 19

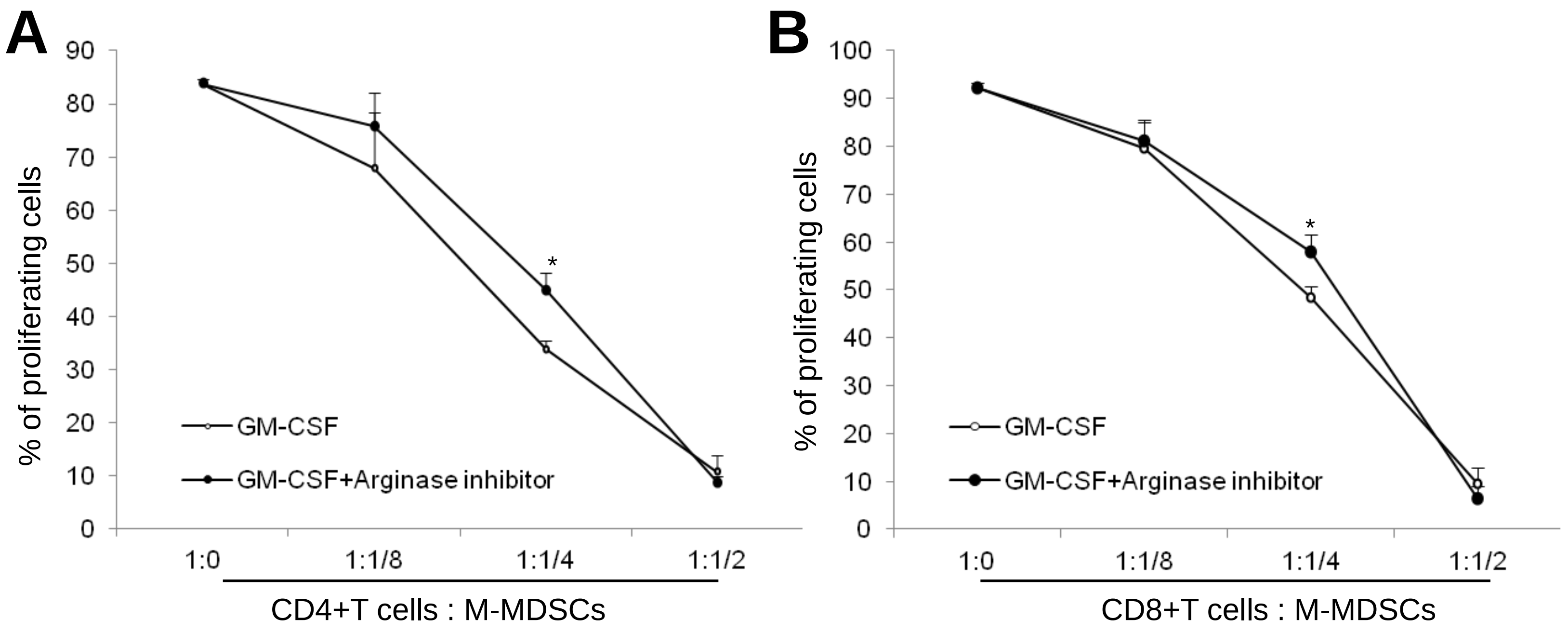

Supplementary figure 19. Arginase inhibitor slightly reversed the immunosuppressive function of M-MDSCs.

GM-CSF-induced M-MDSCs were co-cultured with T cells in the presence of anti-CD3/28 mAbs, 1mM nor-NOHA was added into the T cell suppression assay for 72h. CFSE signals of gated $CD4^+$ (**A**) and $CD8^+$ lymphocytes (**B**) was analyzed.

Suppl. Fig. 20

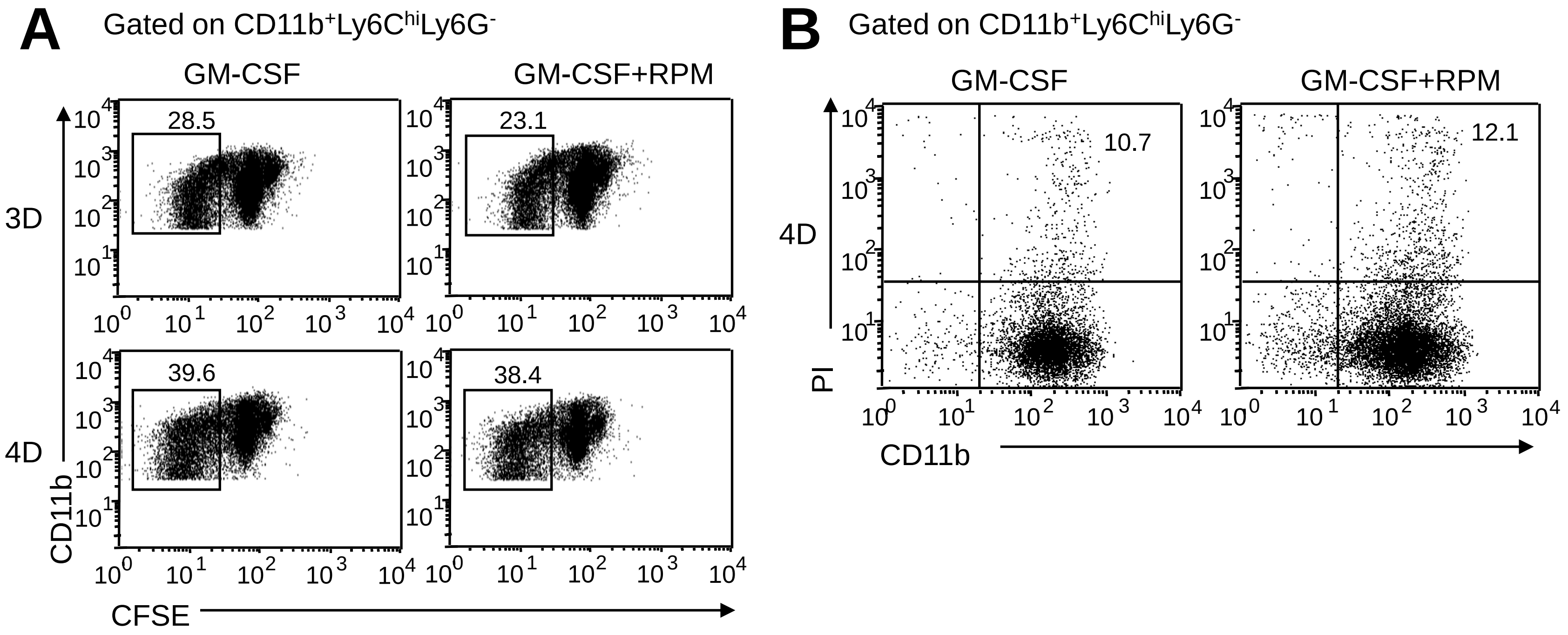

Supplementary figure 20. RPM does not have significant effect on the proliferation and apoptosis of M-MDSCs.

C57BL/6 bone marrow cells were labeled with CFSE and cultured in the presence of 40 ng/ml GM-CSF and 1μM RPM. **(A)**. CFSE signals of gated CD11b^{+}Ly6C^{hi} Ly6G^{-} cells at days 3 and 4 were analyzed. **(B)**. PI staining of gated CD11b^{+}Ly6C^{hi} Ly6G^{-} cells at day 4 after culture was analyzed.

Suppl. Fig. 21

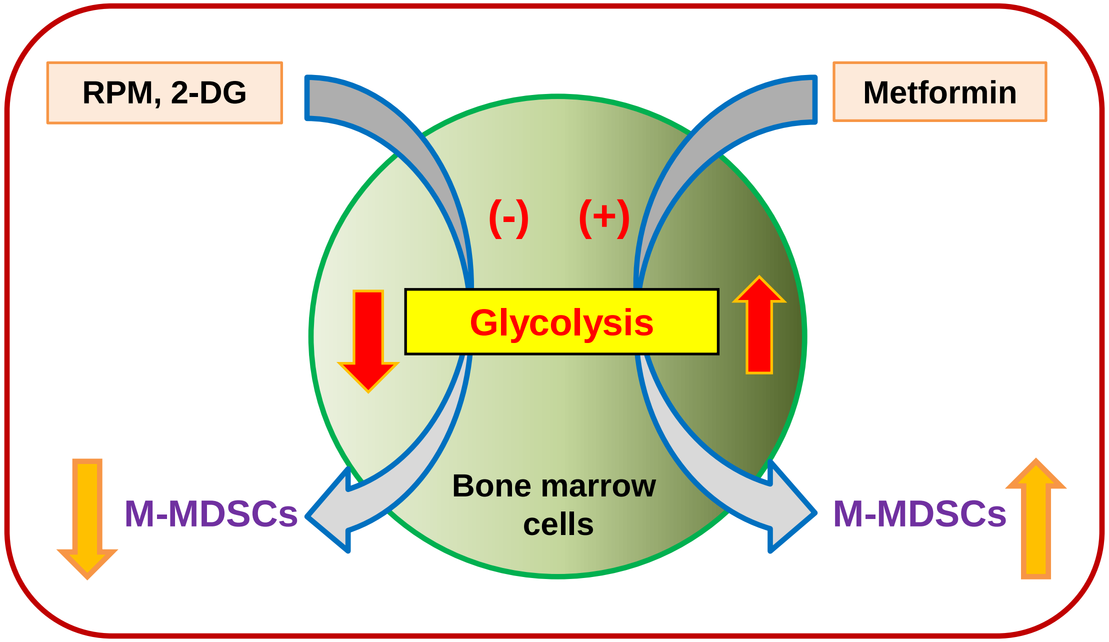

Supplementary figure 21. The different effects of RPM, 2-DG and metformin on the differentiation of M-MDSCs.

RPM and 2-DG inhibit M-MDSC differentiation through decreasing glycolysis pathways, while metformin promotes M-MDSC differentiation through enhancing glycolysis pathways. Cell metabolism states orchestrate the development of M-MDSCs from bone marrow cells, in which glycolysis favors M-MDSC lineage commitment.
